# Ectopic JAK–STAT activation enables the transition to a stem-like and multilineage state conferring AR-targeted therapy resistance

**DOI:** 10.1038/s43018-022-00431-9

**Published:** 2022-09-05

**Authors:** Su Deng, Choushi Wang, Yunguan Wang, Yaru Xu, Xiaoling Li, Nickolas A. Johnson, Atreyi Mukherji, U-Ging Lo, Lingfan Xu, Julisa Gonzalez, Lauren A. Metang, Jianfeng Ye, Carla Rodriguez Tirado, Kathia Rodarte, Yinglu Zhou, Zhiqun Xie, Carlos Arana, Valli Annamalai, Xihui Liu, Donald J. Vander Griend, Douglas Strand, Jer-Tsong Hsieh, Bo Li, Ganesh Raj, Tao Wang, Ping Mu

**Affiliations:** 1 Department of Molecular Biology, UT Southwestern Medical Center, Dallas, TX USA; 2 Quantitative Biomedical Research Center, Department of Population and Data Sciences, UT Southwestern Medical Center, Dallas, TX USA; 3 Department of Urology, UT Southwestern Medical Center, Dallas, TX USA; 4 Department of Pathology, Duke University School of Medicine, Durham, NC USA; 5 Lyda Hill Department of Bioinformatics, UT Southwestern Medical Center, Dallas, TX USA; 6 Department of Neuroscience, UT Southwestern Medical Center, Dallas, TX USA; 7 Wakeland Genomics Core, UT Southwestern Medical Center, Dallas, TX USA; 8 Department of Pathology, The University of Illinois at Chicago, Chicago, IL USA; 9 Hamon Center for Regenerative Science and Medicine, UT Southwestern Medical Center, Dallas, TX USA; 10 Harold C. Simmons Comprehensive Cancer Center, UT Southwestern Medical Center, Dallas, TX USA

**Keywords:** Prostate cancer, Cancer therapeutic resistance, Targeted therapies, Tumour heterogeneity, Cancer

## Abstract

Emerging evidence indicates that various cancers can gain resistance to targeted therapies by acquiring lineage plasticity. Although various genomic and transcriptomic aberrations correlate with lineage plasticity, the molecular mechanisms enabling the acquisition of lineage plasticity have not been fully elucidated. We reveal that Janus kinase (JAK)–signal transducer and activator of transcription (STAT) signaling is a crucial executor in promoting lineage plasticity-driven androgen receptor (AR)-targeted therapy resistance in prostate cancer. Importantly, ectopic JAK–STAT activation is specifically required for the resistance of stem-like subclones expressing multilineage transcriptional programs but not subclones exclusively expressing the neuroendocrine-like lineage program. Both genetic and pharmaceutical inhibition of JAK–STAT signaling resensitizes resistant tumors to AR-targeted therapy. Together, these results suggest that JAK–STAT are compelling therapeutic targets for overcoming lineage plasticity-driven AR-targeted therapy resistance.

## Abstract (web summary)

Mu and colleagues describe a JAK–STAT signaling-dependent transition to a stem-like, multilineage state that is resistant to prostate cancer AR therapy and propose combinatorial targeting of JAK–STAT proteins to resensitize resistant tumors.

## Main

Despite the clinical success surrounding targeted therapies directed toward driver oncogenes, resistance to these therapies often emerges quickly, resulting in poor clinical outcomes. One of the most salient examples of this phenomenon is metastatic castration-resistant prostate cancer (mCRPC), whereby resistance to androgen receptor (AR)-targeted therapies occurs rapidly, and subsequent disease progression is often inevitable^1^. Several mechanisms have been revealed to confer resistance to AR-targeted therapy, such as restoration of the AR-driven transcriptional program or bypass of AR signaling through the activation of other transcription factors^1^. Emerging evidence has demonstrated a third mechanism called lineage plasticity, whereby luminal prostate epithelial cells transition to a lineage-plastic state where survival is no longer dependent on AR^2^. The acquisition of lineage plasticity may result in cells transitioning to a stem cell-like and multilineage state followed by redifferentiation to new lineages or possibly direct transdifferentiation to a different lineage, such as a neuroendocrine (NE)-like lineage^2^.

Lineage plasticity has been observed in mCRPC and is characterized by various genomic and transcriptional aberrations^3–13^, which parallels examples documented in *EGFR*-mutant lung adenocarcinoma, estrogen receptor-positive breast cancers and *BRAF*-mutant melanoma^14–16^. One example of lineage plasticity-driven resistance occurs in mCRPC with concurrent loss of function of TP53 and RB1, which is then accompanied by ectopic activation of SOX2 (refs. ^4,5,17,18^). However, the molecular mechanism that promotes lineage plasticity in many mCRPC subtypes, especially in the context of TP53/RB1 deficiency, is not fully understood. Furthermore, although heterogenous subpopulations have been connected to prostate cancer (PCa) progression and AR therapy resistance^19–24^, the key survival factor of lineage-plastic and stem-like cells has yet to be defined. Finally, therapeutic approaches targeting lineage plasticity-driven resistance are not currently available, underscoring the unmet clinical urgency to identify druggable targets that drive lineage plasticity.

Here, we reveal that the ectopic activation of Janus kinase (JAK)–signal transducer and activator of transcription (STAT) signaling is required for lineage plasticity-driven AR-targeted therapy resistance in mCRPC with TP53/RB1 deficiency and SOX2 upregulation. Single-cell RNA-sequencing (scRNA-seq) analysis revealed that JAK–STAT signaling is specifically required for AR therapy resistance of subclones expressing stem-like and multilineage transcriptional programs but not for AR therapy resistance of subclones exclusively expressing the NE-like lineage program. We demonstrate that both genetic and pharmaceutical inactivation of key components of the JAK–STAT pathway, including JAK1/JAK2 and STAT1/STAT3, resensitize resistant mCRPC to AR-targeted therapy. Collectively, these findings suggest that JAK–STAT signaling is a crucial executor driving lineage plasticity and could be a potential therapeutic target designed to overcome AR-targeted therapy resistance.

## Results

### The JAK–STAT pathway is altered concomitantly with TP53, RB1 and SOX2

**Fig. 1:**
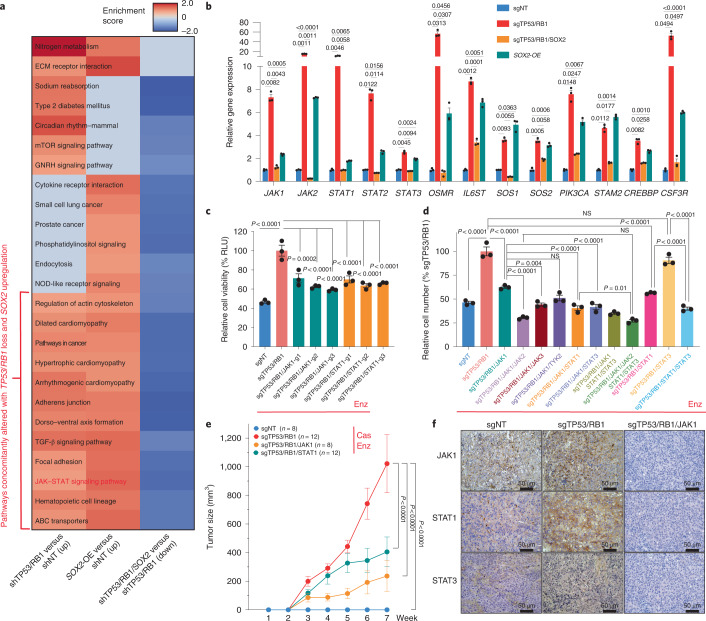
JAK–STAT signaling is required for Enz resistance in TP53/RB1-deficient mCRPC. **a**, Heat map representing the significantly changed signaling pathways in LNCaP/AR cell lines transduced with annotated shRNAs based on GSEA analysis. Three comparisons are presented. Reads from *n* = 3 independently treated cell cultures in each group were used for analysis. Signaling pathways concomitantly altered with *TP53*/*RB1* loss and *SOX2* upregulation are labeled with a red bracket. **b**, Relative gene expression of canonical genes activated in the JAK–STAT signaling pathway in LNCaP/AR cells transduced with Cas9 and annotated guide RNAs; *n* = 3 independently treated cell cultures. *P* values were calculated using a two-way ANOVA with a Bonferroni multiple-comparison test. **c**, Relative cell numbers of LNCaP/AR cells transduced with Cas9 and annotated CRISPR guide RNAs. Cells were treated with 10 µM Enz for 8 d, and cell numbers (viability) were measured using a CellTiter-Glo assay, with all values normalized to the sgTP53/RB1 group; *n* = 3 independently treated cell cultures. *P* values were calculated by one-way ANOVA with a Bonferroni multiple-comparison test; RLU, relative light units. **d**, Relative cell numbers of LNCaP/AR cells transduced with Cas9 and annotated CRISPR guide RNAs. Cells were treated with 10 µM Enz for 8 d, and cell numbers (viability) were measured using a CellTiter-Glo assay, with all values normalized to the sgTP53/RB1 group; *n* = 3 independently treated cell cultures. *P* values were calculated by one-way ANOVA with a Bonferroni multiple-comparison test; NS, not significant. **e**, Tumor growth curve of xenografted LNCaP/AR cells transduced with Cas9 and annotated guide RNAs in castrated mice. Cas denotes castration 2 weeks before grafting. Enz denotes Enz treatment at 10 mg kg^–1^ from day 1 of grafting; *n* = number of independent xenografted tumors in each group (two tumors per mouse); sgNT, *n* = 8 tumors; sgTP53/RB1, *n* = 12 tumors; sgTP53/RB1/JAK1, *n* = 8 tumors; sgTP53/RB1/STAT1, *n* = 12 tumors. *P* values were calculated by two-way ANOVA with a Bonferroni multiple-comparison test. **f**, IHC staining of JAK–STAT proteins on annotated xenografted tumor slides showing representative images of *n* = 2 independent tumors. Source data

**Extended Data Fig. 1:**
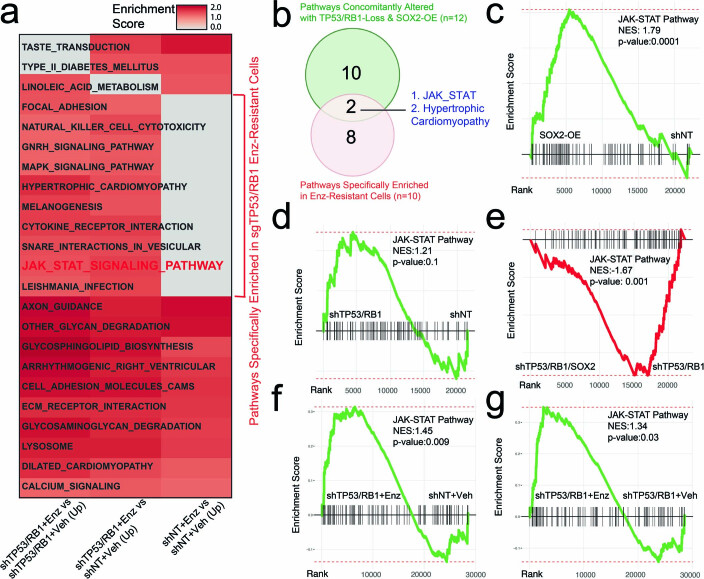
JAK–STAT signaling pathway is enriched in Enz resistant mCRPC with TP53/RB1-deficiency. a, Heat map represents the significantly changed signaling pathways in LNCaP/AR cell lines transduced with annotated shRNAs and treated with Enz or vehicle, based on GSEA analysis. Signaling pathways specifically enriched in shTP53/RB1 Enz-resistant cells are labeled with red bracket. Three comparations are presented and reads from n = 3 independently treated cell cultures were used for analysis. b, Venn diagram represents the signaling pathways concomitantly altered with TP53/RB1-Loss and SOX2-OE, while also specifically enriched in in shTP53/RB1 Enz-resistant cells. c-g, GSEA analysis of JAK–STAT signaling pathway (KEGG_JAK_STAT_Signaling_Pathway) expression in: (c) SOX2-OE group compared to shNT group; (d) shTP53/RB1 group compared to shNT group; (e) shTP53/RB1 group compared to shTP53/RB1/SOX2 group; (f) shTP53/RB1 + Enz group compared to shNT-Veh group; (g) shTP53/RB1 + Enz group compared to shTP53/RB1 + Veh group. For panel c-g, reads from n = 3 independently treated cell cultures were used for analysis. GSEA p-values were calculated with two-sided permutation test by simulations.

To investigate the mechanisms of lineage plasticity in TP53/RB1-deficient mCRPC with SOX2 upregulation, we first inquired which transcriptional programs were altered concomitantly with both the loss of TP53 and RB1 and the upregulation of SOX2. By leveraging a series of LNCaP/AR cell lines we have previously– generated^5^, we profiled transcriptomic changes induced by TP53/RB1 deficiency and overexpression of *SOX2* in four cell lines (control non-targeting short hairpin RNA (shNT), shTP53/RB1, shTP53/RB1/SOX2 and *SOX2* overexpression (*SOX2*-OE)) before exposure to the AR therapy drug enzalutamide (Enz)^25^. As expected, these genetic modifications led to global transcriptomic changes, and gene set enrichment analysis (GSEA) revealed significantly altered pathways (Fig. 1a and Supplementary Tables 1–6), including the duality of specific pathways, where they demonstrated upregulation in *TP53/RB1* double-knockdown (shTP53/RB1) and *SOX2*-OE cells and, by contrast, downregulation in *TP53*/*RB1*/*SOX2* triple-knockdown cells (Fig. 1a). To further decipher which of these transcriptional changes specifically contribute to AR therapy resistance, we investigated signaling pathways enriched following treatment with Enz compared to vehicle (Extended Data Fig. 1a and Supplementary Tables 1–6). Notably, the JAK–STAT signaling pathway was the sole cancer-related pathway that was concomitantly altered with *TP53*/*RB1* loss and *SOX2* upregulation (Extended Data Fig. 1b) and was also consistently upregulated in the sgTP53/RB1 Enz-resistant cells (Extended Data Fig. 1c–g). Interestingly, the JAK–STAT pathway was not significantly altered in shNT cells treated with Enz compared to cells treated with vehicle, suggesting that the JAK–STAT pathway has a specific role in the context of TP53/RB1 deficiency (Extended Data Fig. 1a).

JAK–STAT signaling regulates various biological processes, such as embryonic development, immune response, inflammation, cell fate decision, differentiation and hematopoiesis^26,27^. Notably, numerous lines of evidence implicate JAK–STAT signaling in the regulation of stem cell self-renewal and multilineage differentiation^28^. The consequence of JAK–STAT activation on tumorigenesis is complicated and considered a ‘double-edged sword’. On one hand, JAK–STAT signaling promotes antitumor immune surveillance and therapy-induced cell death and is associated with a favorable clinical outcome in various cancers^29,30^. On the other hand, constitutive activation of JAK–STAT signaling has been correlated with poor clinical outcomes in hematological malignancies and many solid tumors, including PCa^31–42^. In addition, JAK–STAT activation promotes epithelial-to-mesenchymal transition (EMT), invasion and metastasis of PCa^43–47^, further indicating its role in regulating PCa lineage transition. Thus, the observed ectopic upregulation of JAK–STAT signaling in the TP53/RB1-deficient and *SOX2*-OE PCa cells raises the intriguing possibility that it may play a crucial role in acquiring lineage plasticity-driven AR therapy resistance.

### JAK–STAT signaling is required for lineage plasticity and resistance

**Extended Data Fig. 2:**
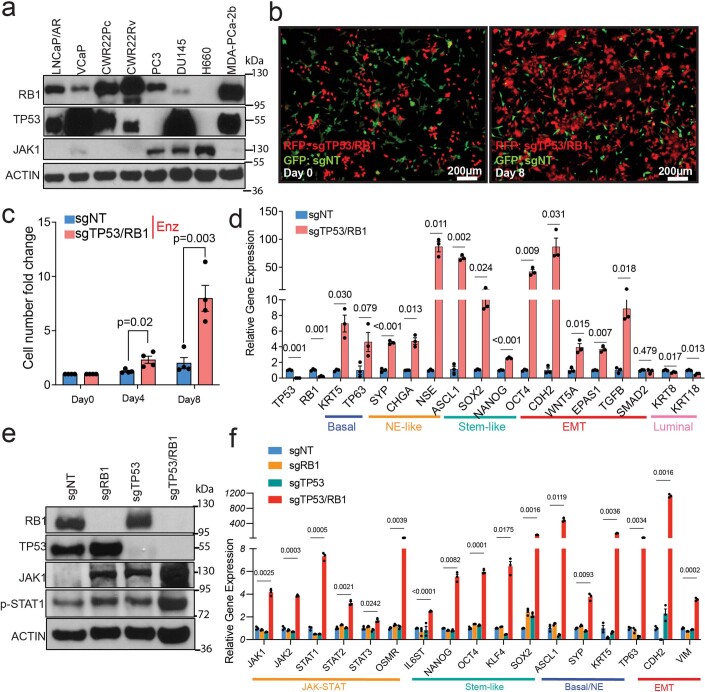
LNCaP/AR-sgTP53/RB1 is a highly resistant and lineage plastic cell line model. a, Western Blot represents the level of RB1, TP53, JAK1 and ACTIN proteins in a series of human PCa cell line models; representative pictures of 2 repeats with similar results were shown. b, Fluorescence microscope imaging shows the cell mixtures of sgTP53/RB1-RFP cells (red) and sgNT-GFP cells (green) on Day 0 and Day 8 of the competition assay cultured in CSS medium and 10 µM Enz; representative pictures of n = 4 independently treated cell cultures were shown. c, Relative cell number fold change of LNCaP/AR cells transduced with Cas9 and annotated guide RNAs measured in the competition assay. The cell mixtures of sgNT-GFP and sgTP53/RB1-RFP were treated in CSS medium with 10 µM Enz for 8 days and the number of GFP/RFP positive cells were measured by FACS on Day 0, 4 and 8. n = 4 independently treated cell cultures. d, Relative expression of canonical lineage marker genes in LNCaP/AR cells transduced with Cas9 and annotated guide RNAs. For panel (c-d), p-values were calculated using two-tailed multiple t-test with Welch’s correction and annotated in figure. e, Western Blot represents the level of RB1, TP53, JAK1, p-STAT1 and ACTIN proteins in LNCaP/AR cells transduced with Cas9 and annotated guide RNAs; representative of 2 repeats with similar results were shown. f, Relative expression of canonical JAK–STAT signaling and lineage marker genes in LNCaP/AR mCRPC cells transduced with Cas9 and annotated guide RNAs. p-values were calculated using two-way ANOVA with Bonferroni multiple-comparison test and annotated in figure. For all panels unless otherwise noted, n = 3 independently treated cell cultures and mean ± s.e.m. is represented. Source data

To examine the role of JAK–STAT signaling in Enz resistance, we first surveyed a series of PCa cell lines and determined the protein levels of TP53, RB1 and JAK1. Here, we observed a substantial accumulation of JAK1 in all three Enz-resistant cell lines (DU145, PC3 and H660; Extended Data Fig. 2a), which are all characterized by TP53/RB1 deficiency (deletion/mutation), compared to in Enz-sensitive cell lines (LNCaP/AR, CWR22Pc, MDA-PCa-2b, VCaP and CWR22Rv). To further dissect the role of JAK–STAT signaling, we generated a stable sgTP53/RB1 clone by knocking out *TP53* and *RB1* in LNCaP/AR cells with CRISPR guides *cis* linked with red fluorescent protein (RFP). These sgTP53/RB1 cells proliferated significantly quicker after exposure to Enz than sgNT cells expressing green fluorescent protein (GFP; Extended Data Fig. 2b,c and Supplementary Fig. 1). sgTP53/RB1 cells displayed clear lineage plasticity, as they express significantly decreased levels of luminal lineage genes and increased levels of non-luminal lineage genes (Extended Data Fig. 2d). We also observed significant upregulation in the expression of canonical JAK–STAT signaling genes in sgTP53/RB1 cells, which was comparable to the levels of JAK–STAT signaling genes induced by *SOX2* OE (Fig. 1b). Interestingly, only double knockout (KO) of *TP53*/*RB1*, but not individual KO of either *TP53* or *RB1*, led to significant JAK–STAT activation and lineage plasticity (Extended Data Fig. 2e,f), suggesting that TP53 and RB1 cooperatively suppress ectopic JAK–STAT activation.

**Extended Data Fig. 3:**
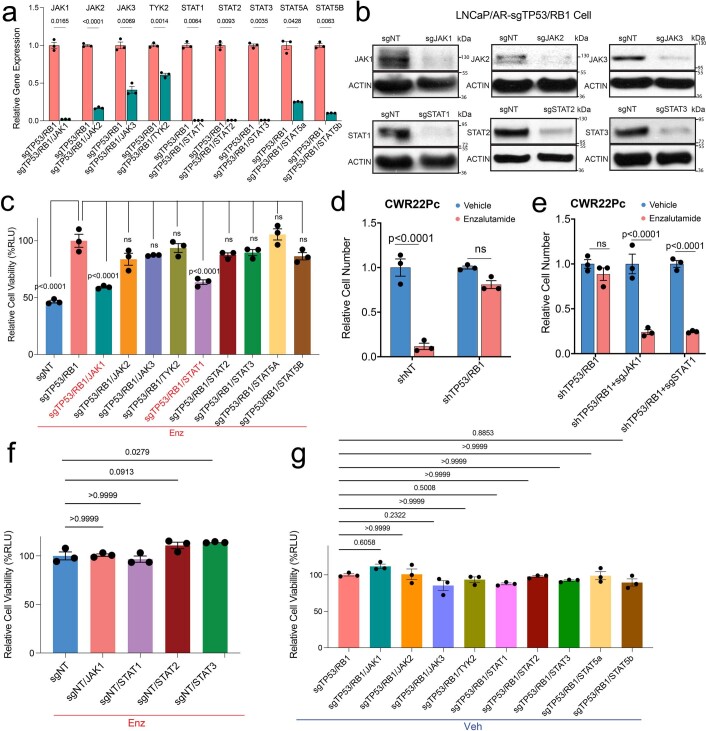
JAK–STAT signaling is specifically required for AR therapy resistance. a, Relative expression of JAK–STAT genes in LNCaP/AR-sgTP53/RB1 cells transduced with Cas9 and annotated guide RNAs. p-values were calculated using two-tailed multiple t-test with Welch’s correction. b, Western blot of JAK1-3 and STAT1-3 proteins in LNCaP/AR cells transduced with Cas9 and annotated guide RNAs; representative pictures of 2 repeats with similar results were shown. c, Relative cell number of LNCaP/AR cells transduced with annotated CRISPR guide RNAs. Cells were treated with 10 µM enzalutamide (Enz) for 8 days, and cell numbers (viability) were measured using CellTiter-Glo assay, all normalized to sgTP53/RB1 group. p-values were calculated using one-way ANOVA with Bonferroni multiple-comparison test. d, Relative cell number of CWR22Pc cells transduced with annotated shRNAs. e, Relative cell number of CWR22Pc cells transduced with annotated shRNAs and/or Cas9 and CRISPR guide RNAs. For panel d-e, cell numbers were measured by cell proliferation assay, normalized to Veh condition, and p-values were calculated using two-way ANOVA with Bonferroni multiple-comparison. f, Relative cell number of LNCaP/AR-sgNT cells transduced with Cas9 annotated CRISPR guide RNAs. Cells were treated with 10 µM enzalutamide (Enz) for 8 days and cell number was measured using CellTiter-Glo assay, all normalized to sgNT group. p-values were calculated using one-way ANOVA with Bonferroni multiple-comparison test. g, Relative cell number of LNCaP/AR-sgTP53/RB1 cells transduced with Cas9 annotated CRISPR guide RNAs. Cells were treated with DMSO for 8 days and cell number was measured using CellTiter-Glo assay, all normalized to sgTP53/RB1 group. p-values were calculated using one-way ANOVA with Bonferroni multiple-comparison test. For all panels unless otherwise noted, n = 3 independently treated cell cultures and mean ± s.e.m. is represented. p-values were annotated in figures. Source data

To determine whether sustained JAK–STAT signaling is required to maintain resistance, we knocked out key JAK–STAT signaling genes in sgTP53/RB1 cells and observed that only KO of *JAK1* and *STAT1* significantly blunted resistant growth of sgTP53/RB1 cells (Fig. 1c and Extended Data Fig. 3a–c). However, these results did not preclude the possibility that different JAK and STAT proteins may function within a cooperative network to regulate AR-targeted therapy resistance. Therefore, we knocked out various combinations of JAK and STAT proteins in the *TP53*/*RB1* double-KO cells and observed that KO of *JAK1* and *JAK2* had a significantly more profound effect on inhibiting Enz-resistant growth of PCa cells than KO of *JAK1* alone, suggesting a cooperative function of JAK2 and JAK1 in conferring Enz resistance (Fig. 1d). Similarly, KO of *STAT1* and *STAT3* had a significantly more profound effect on inhibiting Enz-resistant growth than KO of *STAT1* alone (Fig. 1d), demonstrating how STAT3 and STAT1 function cooperatively to regulate resistance. These results were further validated in an additional Enz-sensitive PCa cell line, CWR22Pc (Extended Data Fig. 3d,e). Interestingly, KO of *JAK*–*STAT* genes in wild-type sgNT cells or in sgTP53/RB1-KO cells treated with vehicle did not influence tumor cell survival (Extended Data Fig. 3f,g), suggesting a specific role of JAK–STAT signaling in lineage plasticity-driven AR therapy resistance. These findings were validated in vivo in castrated severe combined immunodeficient (SCID) mice treated with Enz, where the depletion of JAK1 and STAT1 largely resensitized sgTP53/RB1 xenografted tumors to Enz (Fig. 1e,f).

**Fig. 2:**
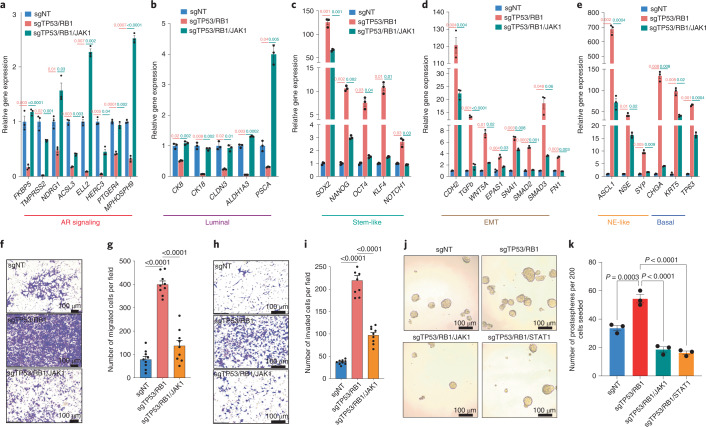
*JAK1* KO stagnates the lineage transition to a stem-like and multilineage state. **a**–**e**, Relative expression of canonical AR target genes and lineage marker genes in LNCaP/AR cells transduced with Cas9 and annotated guide RNAs; *n* = 3 independently treated cell cultures. *P* values were calculated by two-way ANOVA with a Bonferroni multiple-comparison test. **f**, Representative images of an LNCaP/AR cell transwell migration assay of three independent treated cell cultures. **g**, Quantification of the migrated cell numbers of nine representative images taken from three independent treated cell cultures for each of the cell lines. *P* values were calculated by one-way ANOVA with a Bonferroni multiple-comparison test. **h**, Representative images of an LNCaP/AR cell invasion assay of three independent treated cell cultures. **i**, Quantification of the numbers of invading cells of nine representative images taken from three independent treated cell cultures for each of the cell lines. *P* values were calculated by one-way ANOVA with a Bonferroni multiple-comparison test. **j**, Representative images of an LNCaP/AR cell prostasphere formation assay of three independent treated cell cultures. **k**, Quantification of the prostaspheres formed from three independent treated cell cultures for each of the cell lines. *P* values were calculated by one-way ANOVA with a Bonferroni multiple-comparison test. Unless otherwise noted, data are represented as mean ± s.e.m. Source data

**Extended Data Fig. 4:**
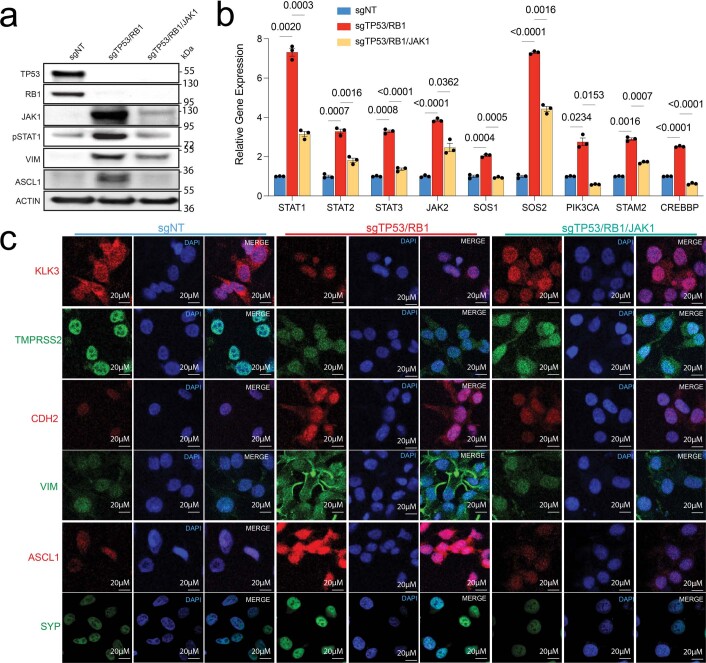
JAK1-KO reversed the acquisition of lineage plasticity. a, Western blot of JAK1, pSTAT1 and lineage marker proteins in LNCaP/AR cells transduced with Cas9 and annotated guide RNAs; representative pictures of 2 repeats with similar results were shown. b, Relative expression of canonical JAK–STAT genes in LNCaP/AR mCRPC cells transduced with Cas9 and annotated guide RNAs. n = 3 independently treated cell cultures and mean ± s.e.m. is represented. p-values were calculated using two-way ANOVA with Bonferroni multiple-comparison test and annotated in figure. c, IF staining of LNCaP/AR cells transduced with Cas9 and annotated guide RNAs with annotated antibodies; representative pictures of n = 2 independent treated cell cultures were shown. Source data

To determine the connection between JAK–STAT signaling and lineage plasticity, we examined the expression of canonical lineage markers in sgTP53/RB1/JAK1 cells, which have suppressed JAK–STAT signaling (Extended Data Fig. 4a,b). We observed that JAK1 depletion largely attenuated the downregulation of AR signaling and the expression of luminal lineage genes (Fig. 2a,b) and upregulation of the expression of stem-like, basal, EMT and NE-like marker genes (Fig. 2c–e), which reinforces its crucial role in the acquisition of non-luminal and multilineage transcriptional programs. Immunofluorescence (IF) staining validated this transition from an exclusively AR-driven luminal lineage to an AR-independent, multilineage state after TP53/RB1 depletion (Extended Data Fig. 4c), which was largely reversed following *JAK1* KO (Extended Data Fig. 4c). *JAK1* KO also reversed the increased migratory and invasive abilities of sgTP53/RB1 cells (Fig. 2f–i), supporting the necessity of JAK–STAT signaling in the maintenance of an EMT lineage program. Furthermore, *JAK1* or *STAT1* KO also reversed the enhanced prostasphere formation of sgTP53/RB1 cells (Fig. 2j,k), which corroborates the role of JAK–STAT signaling in promoting a stem-like state.

### JAK–STAT activation correlates with poor clinical outcomes

**Fig. 3:**
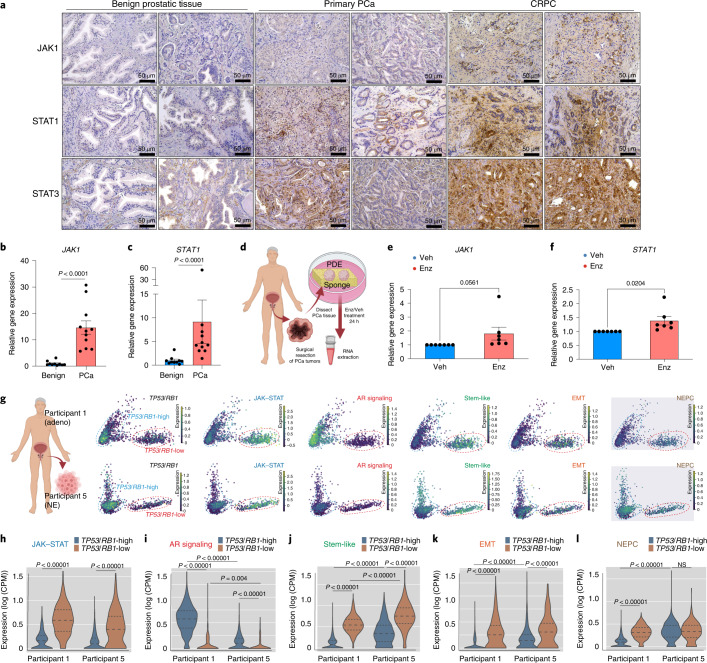
Ectopic JAK–STAT activation correlates with poor clinical outcomes. **a**, IHC staining of annotated JAK–STAT proteins on benign prostate tissues or PCa samples; *n* = 2 independent tumors in each group. **b**,**c**, Relative expression of *JAK1* (**b**) and *STAT1* (**c**) in benign prostate tissues or PCa samples. The center line indicates the median, the box limits indicate upper and lower quartiles and the whiskers indicate maximum and minimum values. *P* values were calculated by a two-sided Mann–Whitney test; *n* = 10 benign prostate samples; *n* = 11 PCa tumors. **d**, Schematic figure representing the generation and examination of the PDE model. The figure was created with BioRender.com; Veh, vehicle. **e**, Relative expression of *JAK1* in a series of PDEs treated with vehicle (DMSO) or Enz (10 µM) for 24 h. **f**, Relative expression of *STAT1* in a series of PDEs treated with vehicle (DMSO) or Enz (10 µM) for 24 h. For **e** and **f**, *n* = 7 independent PDEs, and data show mean ± s.e.m. *P* values were calculated by two-sided *t*-test. **g**, Principal-component analysis (PCA) plots of human CRPC biopsy samples; participant 1, *n* = 2,691 cells, CRPC-adeno; participant 5, *n* = 2,123 cells, CRPC-NE. For each sample, single-cell transcriptomic profiles are colored by the expression (log_2_ CPM) of selected genes representing canonical signaling pathways and lineage-related transcriptional programs. The schematic figure was created with BioRender.com. **h**–**l**, Violin plots representing the expression scores of canonical JAK–STAT signaling, AR signaling and lineage marker genes in subclones with high versus low *TP53*/*RB1* expression in both participants 1 and 5. The center line indicates the median, upper and lower lines indicate upper and lower quartiles and violin limits indicate maximum and minimum values; *TP53*/*RB1*-high: participant 1 *n* = 2,215 cells and participant 5 *n* = 1,796 cells; *TP53*/*RB1*-low: participant 1 *n* = 476 cells and participant 5 *n* = 327 cells. *P* values were calculated by two-sided Mann–Whitney test. Source data

**Extended Data Fig. 5:**
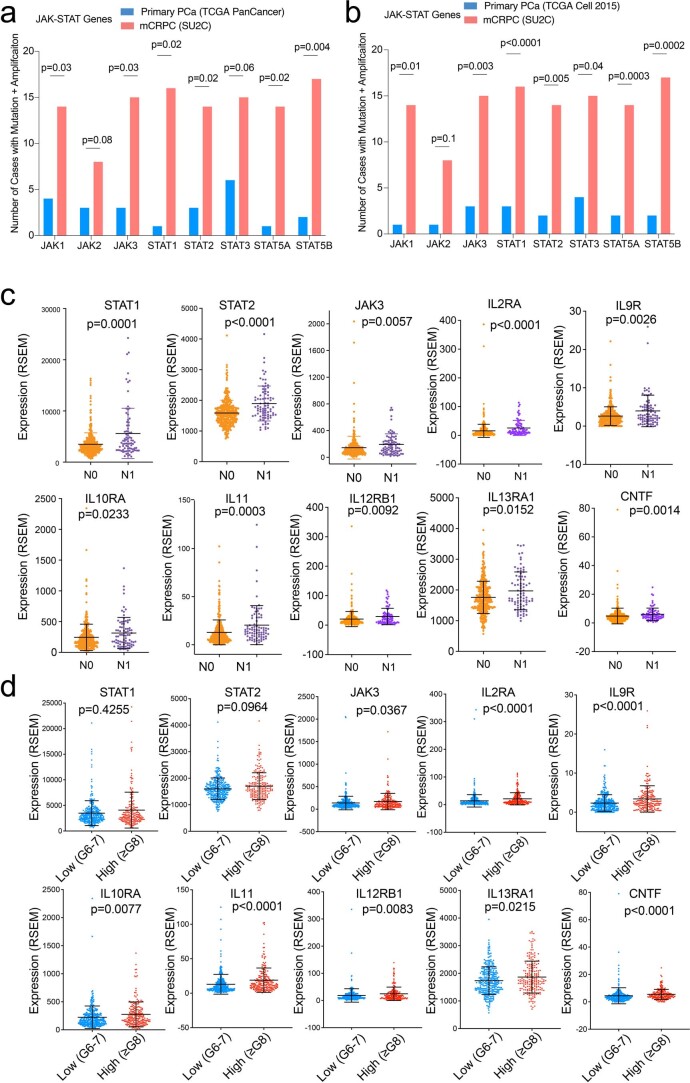
JAK1 and STAT1 genomic alterations is correlated with poor outcome of patients with mCRPC. a, Number of PCa cases with genomic alterations (amplification or mutation) in the loci of key JAK–STAT signaling genes in the mCRPC tumors of the SU2C cohort, compared to the number in the primary tumors of the TCGA PanCancer cohort. TCGA PanCancer = 489 tumors, SU2C = 444 tumors. b, Number of PCa cases with genomic alterations (amplification or mutation) in the loci of key JAK–STAT signaling genes in the mCRPC tumors of the SU2C cohort, compared to the frequency in the primary tumors of the TCGA Cell 2015 cohort. TCGA Cell 2015 = 333 tumors, SU2C = 444 tumors. For panel (a-b), p-values were calculated using two-tails Fisher’s exact test and annotated in figures. c, Expression (RSEM) of JAK–STAT signaling genes in patients with regional lymph nodes metastasis (N1, n = 80 tumors) compared to patients without regional lymph nodes metastasis (N0, n = 345 tumors). d, Expression (RSEM) of JAK–STAT signaling genes in the high-grade tumors (Gleason score ≥ 8, n = 206 tumors) compared to the low-grade tumors (Gleason score ≤ 7, n = 292 tumors). For panel (c-d), mean ± s.d. is represented and p-values were calculated using two-sided Mann–Whitney test and annotated in figures. Source data

Given the prominent role of JAK–STAT signaling in promoting EMT and AR therapy resistance in our preclinical model, we examined the impact of JAK–STAT upregulation in various clinically relevant models and scenarios. We performed immunohistochemistry (IHC) staining of key JAK–STAT proteins in a collection of deidentified human PCa samples and matched benign prostate tissues and validated the substantial augmentation of JAK–STAT signaling in human PCa samples, especially CRPC samples, compared to matched benign tissue (Fig. 3a). Consistent with the IHC results, human PCa tumor samples exhibited a significant enhancement in the expression of JAK1 and STAT1 compared to that observed in benign tissues (Fig. 3b,c). We then treated seven independent human-derived explants (PDE) and observed an upregulation of JAK1 and STAT1 following Enz treatment (Fig. 3d–f)^48,49^, further demonstrating their role in mediating AR therapy resistance. Next, we investigated two human PCa cohorts (The Cancer Genome Atlas (TCGA) and SU2C) and hypothesized that reduced sensitivity to AR-targeted therapy would correlate with a higher frequency of copy number variations of JAK–STAT genes in mCRPC tumors than in hormone-sensitive primary tumors^50–52^. Indeed, the frequencies of copy number amplifications and somatic mutations in JAK–STAT signaling genes were significantly higher in mCRPC (SU2C) than in hormone-naive PCa (TCGA; Extended Data Fig. 5a,b). Finally, we examined both the pathological characteristics and the expression of JAK–STAT signaling genes in the TCGA cohort and discovered that individuals with regional lymph node metastasis (N1) or high-grade tumors (Gleason score of ≥8) had significantly higher JAK–STAT signaling gene expression than individuals lacking regional lymph node metastasis (N0) or with low-grade tumors (Gleason score of ≤7; Extended Data Fig. 5c,d).

**Extended Data Fig. 6:**
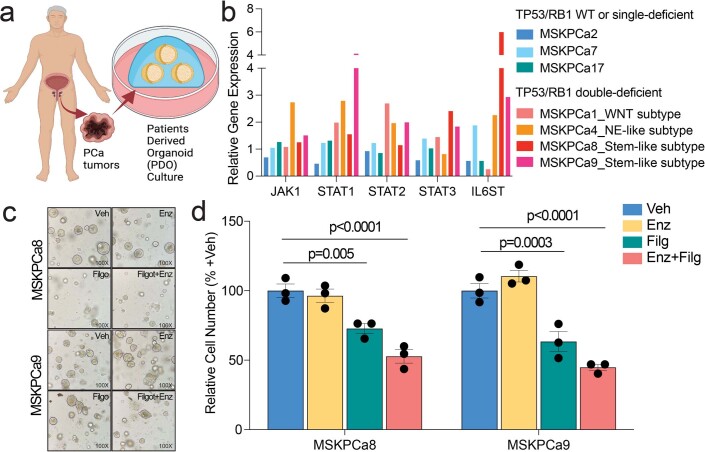
JAK–STAT inhibition reversed the lineage plasticity-driven AR therapy resistance in PDO. a, Schematic figure represents the generation and examination of patient-derived organoid (PDO) model. Figure was created with BioRender.com. b, Relative expression of JAK–STAT genes in a series of PDOs based on RNA-seq results (see Methods). c, Bright field pictures of PDO MSKPCa8 and MSKPCa9 cultured in 3D matrigel and treated with DMSO (Veh), 10 μM enzalutamide (Enz), 5 μM filgotinib (Filg) or the combination of Enz and Filg (Enz + Filg) for 6 days, representative pictures of n = 3 independent treated cell cultures. d, Relative cell number of PDO MSKPCa8 and MSKPCa9 treated with annotated treatments for 6 days, normalized to “Veh” group. Treatment’s denotation is same as panel (c). n = 3 independently treated cell cultures and mean ± s.e.m. is represented. p-values were calculated using two-way ANOVA with Bonferroni multiple-comparison test and annotated in figure. Source data

To determine whether JAK–STAT signaling is specifically upregulated in human PCa with reduced *TP53*/*RB1* expression, we performed transcriptomic analysis of an existing human CRPC scRNA-seq dataset^24^. Among the six individuals of this cohort, we identified two major clusters of PCa cell subpopulations expressing either high or low levels of both *TP53* and *RB1* in participant 1 (CRPC-adeno) and participant 5 (CRPC-NE; Fig. 3g). Transcriptomic analysis revealed increased expression of JAK–STAT signaling genes, such as *JAK1*, *STAT1* and *IL6ST*, in the *TP53*/*RB1*-low subpopulation compared to in the *TP53*/*RB1*-high subpopulation in both individuals (Fig. 3g,h). Strikingly, the *TP53*/*RB1*-low subpopulations displayed substantially higher expression of stem-like (*TACSTD2*, *ATXN1*, *KRT4* and *CD55*) and EMT (*VIM*, *SNAI2* and *CDH11*) gene and lower AR target (*KLK3*, *PTGER4* and *ACSL3*) gene (Fig. 3g,i–k), which is consistent with the role of JAK–STAT signaling in promoting the transition from an AR-dependent state to an AR-independent, multilineage and stem-like state. Interestingly, an increase in NE-like lineage in the *TP53*/*RB1*-low cells was only observed in participant 1 (CRPC-adeno) but not in participant 5 (CRPC-NE; Fig. 3g,l). These data indicate that JAK–STAT may be dispensable for tumor cells exclusively expressing NE-like lineage. To further validate whether ectopic JAK–STAT is required for resistance in human PCa, we surveyed a series of three-dimensional (3D)-cultured human-derived organoid (PDO) models (Extended Data Fig. 6a)^53–55^ and observed ectopic upregulation of JAK–STAT signaling genes in PDOs with TP53/RB1 deficiency (Extended Data Fig. 6b). Among those PDOs, MSKPCa8 and MSKPCa9 belong to a subclass defined by increased stem-like, EMT-like and interferon response-related transcriptional programs^54,55^. Strikingly, JAK–STAT signaling inhibition by the JAK1 inhibitor filgotinib (Filg) largely resensitized these Enz-resistant PDOs (Extended Data Fig. 6c,d), supporting the crucial role of JAK–STAT in mediating AR therapy resistance.

### JAK1 inhibition reverses lineage plasticity and resistance

**Fig. 4:**
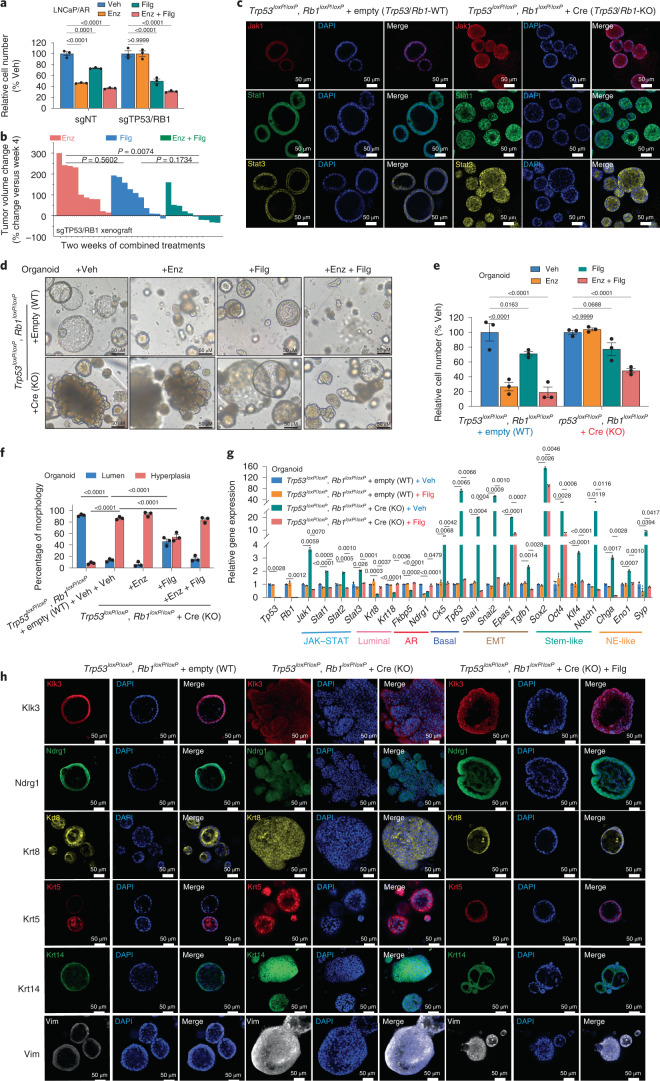
JAK1 inhibitor restores Enz sensitivity. **a**, Relative cell number of LNCaP/AR cells transduced with Cas9 and annotated CRISPR guide RNAs and treated with annotated treatments in CSS medium and normalized to the vehicle group; Enz, 10 μM Enz; Filg, 5 μM Filg; Enz + Filg, combination of Enz and Filg; vehicle, DMSO treatment with equal volume as Enz. Cells were treated for 8 d, and cell numbers were measured by a CellTiter-Glo assay. **b**, Waterfall plot displaying changes in tumor size of xenografted LNCaP/AR-sgTP53/RB1 cells after 2 weeks of treatments. All animals were treated with Enz at 10 mg kg^–1^ orally 1 d after grafting. Beginning from week 3 of xenografting, animals were randomized into three groups and treated with Enz only at 10 mg kg^–1^ orally, Filg only at 20 mg kg^–1^ orally twice daily or a combination of Enz plus Filg; *n* = the number of independent xenografted tumors in each group (two tumors per mouse); Enz, *n* = 10 tumors; Filg, *n* = 10 tumors; Enz + Filg, *n* = 10 tumors. *P* values were calculated by one-way ANOVA with a Bonferroni multiple-comparison test. **c**, IF staining of the *Trp53*^*loxP*/*loxP*^*Rb1*^*loxP*/*loxP*^ + empty (*Trp53*/*RB1*-WT) and *Trp53*^*loxP/*^^l^^*oxP*^*Rb1*^*loxP*^^/^^*loxP*^ + Cre (*Trp53*/*RB1*-KO) organoids in 3D with annotated antibodies; representative images of *n* = 2 independent treated cell cultures are shown. **d**, Brightfield images of annotated organoids treated with DMSO (vehicle), 1 μM Enz, 5 μM Filg or Enz and Filg (Enz + Filg) for 6 d; representative images of *n* = 3 independent treated cell cultures are shown. **e**, Relative cell numbers of annotated organoids treated with annotated treatments for 6 d normalized to the vehicle group. Treatments are the same as described in **d**. **f**, Percentage of organoids that display lumen or hyperplasia morphology. Treatments are the same as described in **d**. **g**, Relative expression of *JAK*–*STAT* and lineage marker genes in organoids treated with the treatments annotated in **d**. **h**, IF staining of the annotated organoids with antibodies targeting the proteins encoded by AR target genes and lineage marker genes; representative images of *n* = 2 independent treated cell cultures are shown. Unless otherwise noted, *n* = 3 independent treated cell cultures, and data represent mean ± s.e.m. *P* values were calculated by two-way ANOVA with a Bonferroni multiple-comparison test. Source data

**Extended Data Fig. 7:**
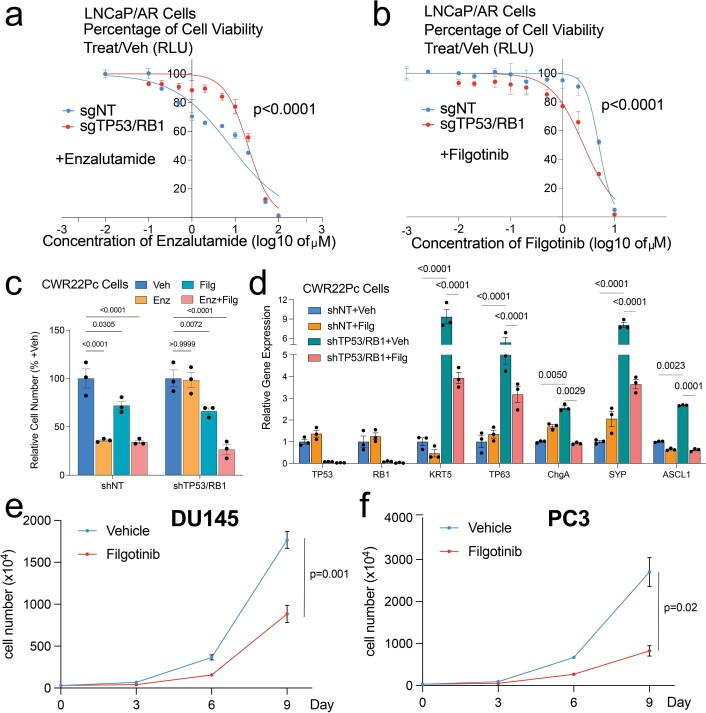
JAK inhibitor impairs lineage plasticity and restores Enz sensitivity. a, Enz dose–response curve of LNCaP/AR cells transduced with Cas9 and annotated CRISPR guide RNAs. b, Filg dose–response curve of LNCaP/AR cells transduced with annotated Cas9 and CRISPR guide RNAs. For panel (a-b), p-values were calculated by non-linear regression with two-sided extra sun-of-squares F test. c, Relative cell number of CWR22Pc cells transduced with annotated shRNAs and treated with various treatments, normalized to “Veh” group. Enz denotes 1 μM Enz, Filg denotes 5 μM filgotinib, Enz + Filg denotes the combination of Enz and Filg and Veh denotes DMSO treatment with equal volume as Enz. Cells were treated for 4 days and cell numbers were counted. d, Relative expression of canonical lineage marker genes in CWR22Pc cells transduced with annotated shRNAs and treated with vehicle or Filg, normalized to “shNT + Veh” group. Filg denotes 5 μM filgotinib, and Veh denotes DMSO treatment with equal volume as Filg. e, Cell number of DU145 cells upon treatment administration, measured by cell proliferation assay. f, Cell number of PC3 cells upon treatment administration, measured by cell proliferation assay. For e-f panels, Filg denotes 5 µM Filgotinib and Veh denotes DMSO treatment for 9 days. For all panels unless otherwise noted, n = 3 independently treated cell cultures and mean ± s.e.m. is represented; p-values were calculated using two-way ANOVA with Bonferroni multiple-comparison test and were annotated in figures. Source data

Identification of JAK–STAT signaling as a crucial executor of lineage plasticity-driven resistance raises the hope that appropriate therapeutic approaches targeting this pathway could overcome AR-targeted therapy resistance. Indeed, in vitro cell viability assays demonstrated that combination treatment of Filg and Enz significantly inhibited the growth of Enz-resistant sgTP53/RB1 LNCaP/AR cells (Fig. 4a). Dose–response measurements (half-maximum inhibitory concentration (IC_50_)) validated that sgTP53/RB1 cells exhibit less sensitivity to Enz than sgNT cells (Extended Data Fig. 7a), while the sgTP53/RB1 cells are more susceptible to Filg than sgNT cells (Extended Data Fig. 7b). These results were again validated in CWR22Pc cells, where Filg significantly inhibited the growth of Enz-resistant cells and attenuated the upregulation of non-luminal lineage programs (Extended Data Fig. 7c,d). Furthermore, Filg impaired the growth of DU145 and PC3 cells, two Enz-resistant PCa cell lines expressing ectopic levels of JAK1 (Extended Data Fig. 7e,f). These in vitro results are further supported by in vivo xenograft experiments, as the combination treatment of Enz and Filg stagnated the growth of Enz-resistant sgTP53/RB1 tumors and induced more tumor regression than either drug alone (Fig. 4b).

**Extended Data Fig. 8:**
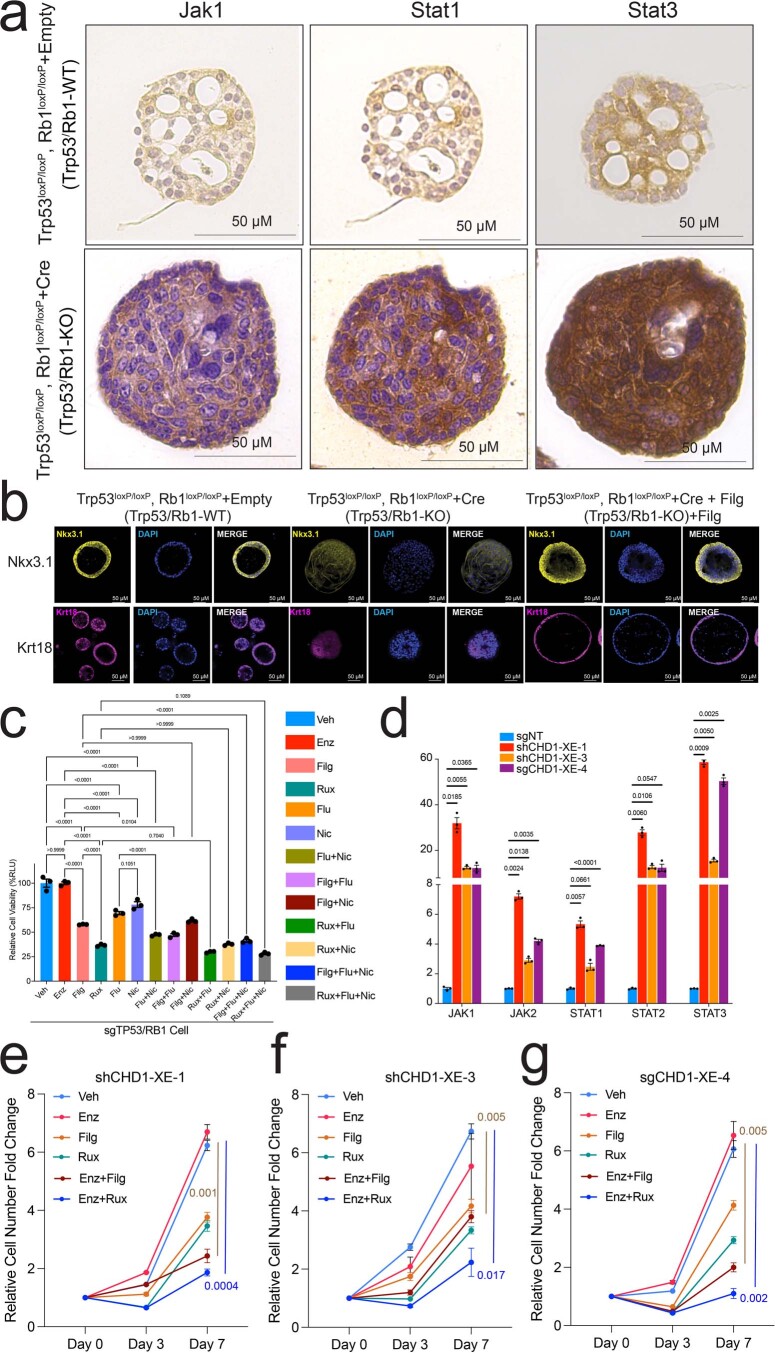
JAK–STAT inhibitors have combined inhibitory effect on PCa cells with lineage plasticity. a, IHC of the Trp53^loxP/loxP^, Rb1^loxP/loxP^ + Empty (TP53/RB1-WT) and Trp53^loxP/loxP^, Rb1^loxP/loxP^ + Cre (TP53/RB1-KO) organoids cultured in 3D, with annotated antibodies. b, IF of annotated organoids cultured in 3D, with annotated antibodies targeting canonical AR target genes and lineage marker genes. For panels a and b, representative pictures of n = 2 independent treated cell cultures were shown. c, Relative cell number of LNCaP/AR cells treated with annotated treatment: 10 µM enzalutamide (Enz), 5 µM filgotinib (Filg), 5 µM ruxolitinib (Ruxo), 1 µM fludarabine (Flu), 0.2 µM niclosamide (Nic) and DMSO for 8 days and cell numbers (viability) were measured using CellTiter-Glo assay, all normalized to vehicle group. p-values were calculated using one-way ANOVA with Bonferroni multiple-comparison test. d, Relative gene expression of JAK–STAT genes in xenograft-derived Enz resistant cell lines with CHD1-deficiency. e-g, Cell number of xenografted-derived Enz resistant cells upon treatment administration, measured by cell proliferation assay. For panels (e-g), Enz denotes 10 µM enzalutamide, Filg denotes 5 µM filgotinib, Rux denotes 5 µM ruxolitinib and Veh denotes DMSO treatment for 7 days. For all panels unless otherwise noted, n = 3 independently treated cell cultures and mean ± s.e.m. is represented. p-values were calculated using two-way ANOVA with Bonferroni multiple-comparison test and annotated in figures. Source data

To further explore the effect of JAK1 inhibition in a genetically defined model, we used the previously established mouse prostate organoids derived from *Trp53*^*loxP*/*loxP*^*Rb1*^*loxP*/*loxP*^ mice, followed by infection with Cre or empty lentivirus^5^. In contrast to the typical lumen structure, which the *Trp53*^*loxP*^^/^^*loxP*^*Rb1*^*loxP*^^/*l*^^*oxP*^ + empty (*Trp53*/*Rb1*-wildtype (WT)) organoids formed in 3D culture, *Trp53*^*loxP*/*loxP*^*Rb1*^*loxP*/*loxP*^ + Cre (*Trp53*/*Rb1*-KO) organoids displayed a hyperplastic morphology, where the organoid cells formed a solid ball with protrusive structures invading the surrounding Matrigel (Fig. 4c,d). The *Trp53*/*Rb1*-KO organoids expressed significantly elevated levels of JAK–STAT proteins compared to *Trp53*/*Rb1*-WT organoids (Fig. 4c and Extended Data Fig. 8a). Although these *Trp53*/*Rb1*-KO organoids were significantly more resistant to Enz than *Trp53*/*Rb1*-WT controls (Fig. 4d,e), they responded well to the combination of Enz and Filg (Fig. 4d,e). Remarkably, we also observed that a substantial number of *Trp53*/*Rb1*-KO organoids reestablished a classic lumen-like structure when treated with Filg (Fig. 4d,f), indicating that JAK1 inhibition impairs the acquisition of non-luminal programs and restores the luminal program. Consistent with this hypothesis, the percentage of lumen-like organoids in the *Trp53*/*Rb1*-KO group significantly receded when treated with Enz and Filg (Fig. 4d,f), suggesting that Enz sensitivity was restored in those lumen-like organoids. The reversal of the lineage plasticity within Filg-treated organoids is supported by quantitative PCR (qPCR) results and IF staining, which demonstrated attenuated downregulation of AR and luminal gene expression and upregulation of non-luminal gene expression (Fig. 4g,h and Extended Data Fig. 8b).

As JAK1/JAK2 and STAT1/STAT3 may cooperatively mediate lineage plasticity and resistance (Fig. 1d), we examined the inhibitory effects of various pharmaceutical inhibitors targeting different JAK and STAT proteins, including ruxolitinib (JAK1/JAK2 inhibitor), fludarabine (STAT1 inhibitor) and niclosamide (STAT3 inhibitor). Interestingly, the dual JAK1/JAK2 inhibitor ruxolitinib had a greater inhibitory effect on *TP53*/*RB1*-KO cells than Filg (Extended Data Fig. 8c). Similarly, combined administration of fludarabine and niclosamide achieved a more profound inhibitory effect on Enz-resistant growth than fludarabine or niclosamide alone (Extended Data Fig. 8c), supporting the cooperative roles of both JAK1/JAK2 and STAT1/STAT3. To further examine whether JAK–STAT signaling mediates lineage plasticity-driven resistance in a broader fashion, we surveyed a series of xenograft-derived, Enz-resistant cell lines with *CHD1* loss, which display clear lineage plasticity^12^, and identified three cell lines with ectopic JAK–STAT signaling (Extended Data Fig. 8d). JAK–STAT inhibition through both Filg and ruxolitinib largely resensitized xenograft-derived resistant cells to Enz (Extended Data Fig. 8e–g), suggesting that PCa cells may hijack JAK–STAT signaling as a general avenue to promote lineage plasticity and resistance.

### SOX2 promotes JAK–STAT signaling in a positive feedback fashion

**Fig. 5:**
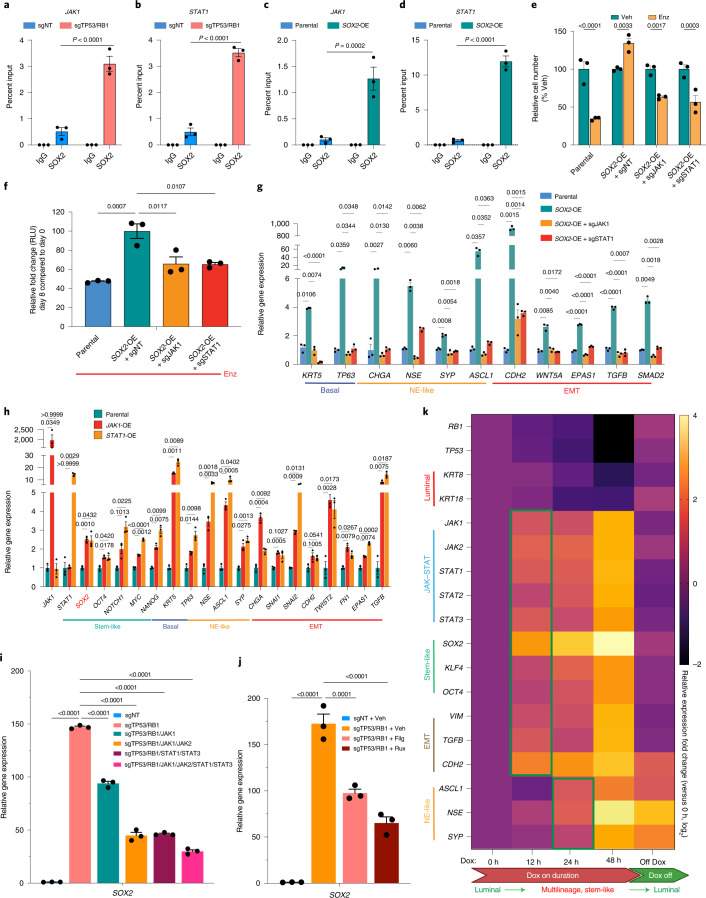
SOX2 enables JAK–STAT activation in a positive feedback fashion. **a**–**d**, SOX2 ChIP–qPCR of *JAK1* (**a**,**c**) and *STAT1* (**b**,**d**) genomic loci in LNCaP/AR cells transduced with annotated CRISPR guide RNAs or overexpressing constructs. **e**, Relative cell numbers of LNCaP/AR cells transduced with annotated constructs and treated with Enz or vehicle, normalized to the vehicle group; Enz, 10 μM Enz; vehicle, DMSO treatment with equal volume as Enz. Cells were treated for 6 d, and cell numbers were measured by cell proliferation assay. **f**, Relative cell number fold change of LNCaP/AR cells transduced with annotated constructs. Data are normalized to the *SOX2*-OE + sgNT group; Enz, 10 μM Enz treatment for 8 d. Cell numbers were measured by a CellTiter-Glo assay. *P* values were calculated by one-way ANOVA with a Bonferroni multiple-comparison test. **g**, Relative expression of canonical lineage marker genes in LNCaP/AR *SOX2*-OE cells transduced with annotated constructs. **h**, Relative expression of canonical lineage marker genes in LNCaP/AR cells transduced with *JAK1* or *STAT1* cDNA constructs. *SOX2* expression is highlighted in red. **i**, Relative expression of *SOX2* in LNCaP/AR cells transduced with annotated guide RNAs. **j**, Relative expression of *SOX2* in LNCaP/AR cells treated with 5 µM Filg or 5 µM ruxolitinib (Rux) or DMSO for 8 d. *P* values in **i** and **j** were calculated by one-way ANOVA with a Bonferroni multiple-comparison test. **k**, Relative gene expression levels of canonical JAK–STAT signaling and lineage marker genes in the inducible shTP53/RB1 LNCaP/AR cells treated with Dox for various lengths of time. Data are normalized to 0 h. Unless otherwise noted, *n* = 3 independent treated cell cultures, and data represent mean ± s.e.m. *P* values were calculated by two-way ANOVA with a Bonferroni multiple-comparison test. Source data

**Extended Data Fig. 9:**
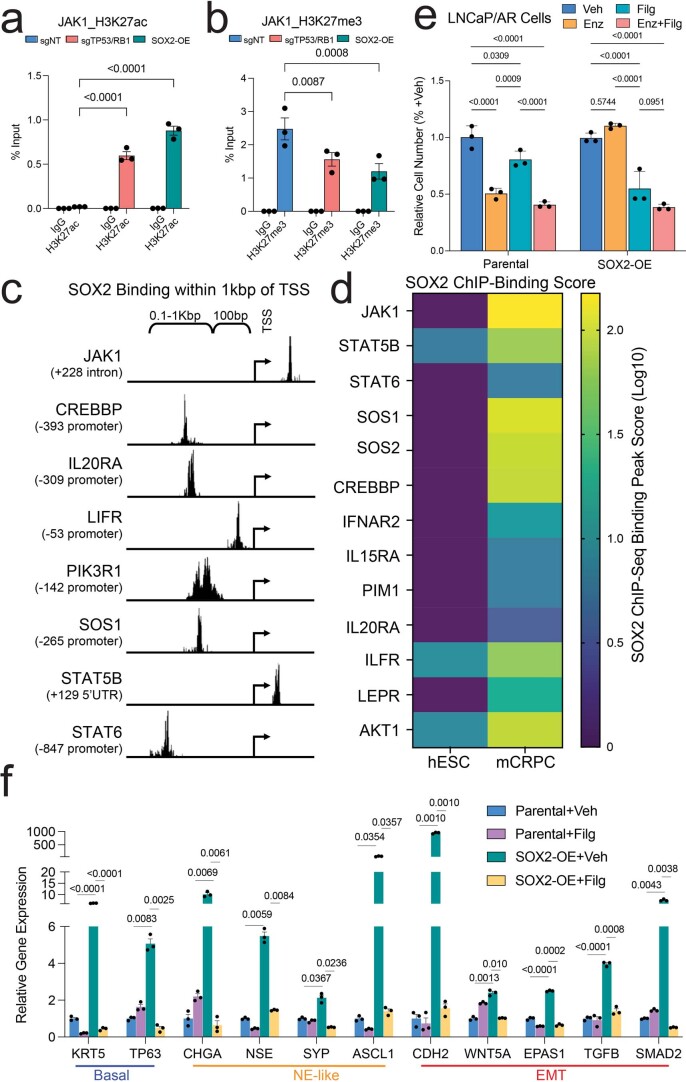
Canonical JAK–STAT genes are among the prostate cancer-specific gene targets of SOX2 in mCRPC. a, H3K27ac ChIP-qPCR of the JAK1 genomic locus in LNCaP/AR cells transduced with annotated constructs. b, H3K27me3 ChIP-qPCR of the JAK1 genomic locus in LNCaP/AR cells transduced with annotated constructs. For panels (a,b), p-values were calculated using one-way ANOVA with Bonferroni multiple-comparison test. c, Representative SOX2 binding sites in the genomic loci of JAK–STAT signaling genes in the mCRPC CWR-R1 cell line based on ChIP-seq analysis. d, SOX2 binding peak score in the genomic loci of JAK–STAT signaling genes in the mCRPC CWR-R1 cell (prostate cancer specific binding) compared to human ESC cell line WA01. Reads from n = 3 independent cell cultures and matching input controls were used for analysis. e, Relative cell numbers of LNCaP/AR cells transduced with annotated constructs and treated with various treatments in CSS medium for 8 days, normalized to “Veh” group. Enz denotes 10 μM enzalutamide, Filg denotes 5 μM filgotinib, Enz + Filg denotes the combination of Enz and Filg, Veh denotes DMSO treatment with equal volume as Enz, for 8 days and cell numbers were counted, normalized to Veh group. f, Relative expression of canonical lineage marker genes in LNCaP/AR-SOX2-OE cells treated with annotated treatments. Filg denotes 5 μM filgotinib, and Veh denotes DMSO treatment with equal volume as Filg. Cells were treated for 6 days. p-values were calculated using two-way ANOVA with Bonferroni multiple-comparison test. For all panels unless otherwise noted, n = 3 independently treated cell cultures and mean ± s.e.m. is represented; p-values were annotated in figures. Source data

We next sought to reveal the mechanism through which JAK–STAT signaling is upregulated. Interestingly, *SOX2* KO in the TP53/RB1-deficient cells impaired the upregulation of JAK–STAT signaling genes (Fig. 1b), indicating a critical role of SOX2 in activation of JAK–STAT signaling. SOX2 chromatin immunoprecipitation (ChIP)–qPCR analysis supports this hypothesis by demonstrating a significant augmentation of SOX2 binding at *JAK*–*STAT* gene loci in cells with *TP53*/*RB1* KO or ectopic SOX2 expression (Fig. 5a–d). Consistent with these SOX2 ChIP–qPCR results, an increase in histone 3 lysine 27 (H3K27) acetylation (H3K27ac) and a decrease in H3K27 trimethylation (H3K27me3) at the *JAK1* gene locus following *TP53*/*RB1* KO or *SOX2* OE were also identified, indicating a transcriptional upregulation of JAK1 by SOX2 (Extended Data Fig. 9a,b). This hypothesis was further supported by analyzing an existing SOX2 ChIP–seq dataset generated from another mCRPC cell line with ectopic SOX2 expression^56^, CWR-R1, which demonstrated PCa-specific SOX2 binding sites in *JAK*–*STAT* genes compared to canonical SOX2 binding sites in the embryonic stem cell line WA01 (Extended Data Fig. 9c,d). To explore whether JAK and STAT are required for SOX2-promoted lineage plasticity and resistance, we knocked out *JAK1* and *STAT1* in the *SOX2*-OE cells and observed significantly impaired resistant growth of those cells, as shown in cell proliferation assays (Fig. 5e) and CellTiter-Glo viability assays (Fig. 5f). Furthermore, *JAK1* and *STAT1* KO in the *SOX2*-OE cells largely attenuated the acquisition of lineage plasticity (Fig. 5g). JAK1 inhibition by Filg significantly resensitized *SOX2*-OE cells to Enz (Extended Data Fig. 9e) and attenuated the acquisition of lineage plasticity in these cells (Extended Data Fig. 9f).

To reveal whether JAK–STAT activation is sufficient to promote lineage plasticity, we overexpressed *JAK1* and *STAT1* (*JAK1*-OE and *STAT1*-OE) in LNCaP/AR cells and observed significantly upregulated expression of stem-like, EMT, basal and NE-like marker genes (Fig. 5h). Notably, the observed upregulation of *SOX2* in *JAK1*-OE and *STAT1*-OE cells (Fig. 5h) suggests positive feedback regulation between SOX2 and JAK–STAT activation. Consistent with this feedback model, JAK1 inhibition through either CRISPR-mediated KO or Filg treatment in sgTP53/RB1 cells led to a ~30% reduction of *SOX2* expression (Fig. 5i,j). Furthermore, combination of KO or pharmaceutical inhibition of various JAK and STAT proteins led to a more profound downregulation of *SOX2* expression (Fig. 5i,j), suggesting that various JAK and STAT proteins cooperatively regulate SOX2 in a similar feedback fashion. Finally, to further decipher the dynamic of this SOX2- and JAK–STAT-regulated lineage plasticity, we used an inducible shRNA-transduced LNCaP/AR model, where doxycycline (Dox)-inducible *TP53*/*RB1* knockdown led to upregulation of JAK–STAT signaling genes as soon as 12 h following Dox administration (Fig. 5k). Remarkably, stem-like and EMT-like programs were spontaneously upregulated with JAK–STAT signaling as soon as 12 h after Dox induction, while NE-like programs were not upregulated until 24 h after Dox administration (Fig. 5k). Furthermore, although stem-like and EMT-like programs were simultaneously reversed to wild-type levels following the downregulation of JAK–STAT signaling after Dox removal, NE-like programs were not fully restored (Fig. 5k), suggesting that NE-like programs were retained in a subset of cells. These results may suggest that JAK–STAT signaling is required for therapy resistance of stem-like and multilineage cells rather than cells exclusively expressing NE-like lineage.

### Single-cell transcriptomics reveal lineage heterogeneity

**Fig. 6:**
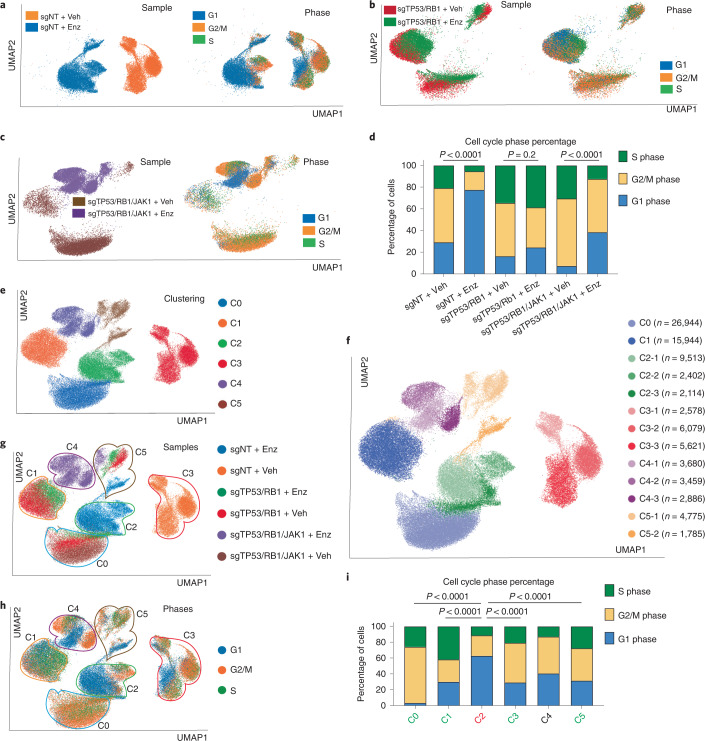
JAK–STAT is required for AR therapy resistance of heterogenous subclones. **a**–**c**, UMAP plots of single-cell transcriptomic profiles of LNCaP/AR cells transduced by annotated CRISPR guide RNAs and treated with vehicle (DMSO) or 10 µM Enz for 5 d; sgNT (Veh, *n* = 14,268 cells; Enz, *n* = 15,149 cells; **a**); sgTP53/RB1 (Veh, *n* = 12,267 cells; Enz, *n* = 9,850 cells; **b**); sgTP53/RB1/JAK1 (Veh, *n* = 25,200 cells; Enz, *n* = 11,096 cells; **c**). Cells on the left are colored according to sample origin, while cells on the right are colored by predicted cell cycle phase. **d**, Bar plot presenting the percent distribution of single cells in different cell cycle phases in each sample. The numbers of cells (*n*) are the same as in **a**–**c**. *P* values were calculated by two-sided Fisher’s exact test. **e**, Single-cell profile of LNCaP/AR cells based on clustering. A UMAP plot of single cells colored by unsupervised clustering of six subsets is presented; cluster 0 (C0), *n* = 26,944 cells; C1, *n* = 15,994 cells; C2, *n* = 14,029 cells; C3, *n* = 14,278 cells; C4, *n* = 10,025 cells; C5, *n* = 6,560 cells. **f**, Single-cell profile of LNCaP/AR cells based on subclustering. A UMAP plot of single cells colored by unsupervised clustering of 13 subclusters is presented; C0, *n* = 26,944 cells; C1, *n* = 15,994 cells; C2-1, *n* = 9,513 cells; C2-2, *n* = 2,402 cells; C2-3, *n* = 2,114 cells; C3-1, *n* = 2,578 cells; C3-2, *n* = 6,079 cells; C3-3, *n* = 5,621 cells; C4-1, *n* = 3,680 cells; C4-2, *n* = 3,459 cells; C4-3, *n* = 2,886 cells; C5-1, *n* = 4,775 cells; C5-2, *n* = 1,785 cells. **g**, Single-cell profile of LNCaP/AR cells transduced with annotated CRISPR guide RNAs and treated with vehicle or Enz. A UMAP plot of single cells colored by samples is represented. The area and number of clusters in **e** are highlighted with colored circles. **h**, Single-cell profile of LNCaP/AR cells based on cell cycle states. A UMAP plot of single cells colored by cell cycle prediction is presented. The area and number of clusters in **e** are highlighted with colored circles. **i**, Bar plot presenting the percent distribution of single cells in different cell cycle phases in each of the six clusters. The number of cells (*n*) in each sample is the same as in **e**. *P* values were calculated by two-sided Fisher’s exact test. Source data

**Extended Data Fig. 10:**
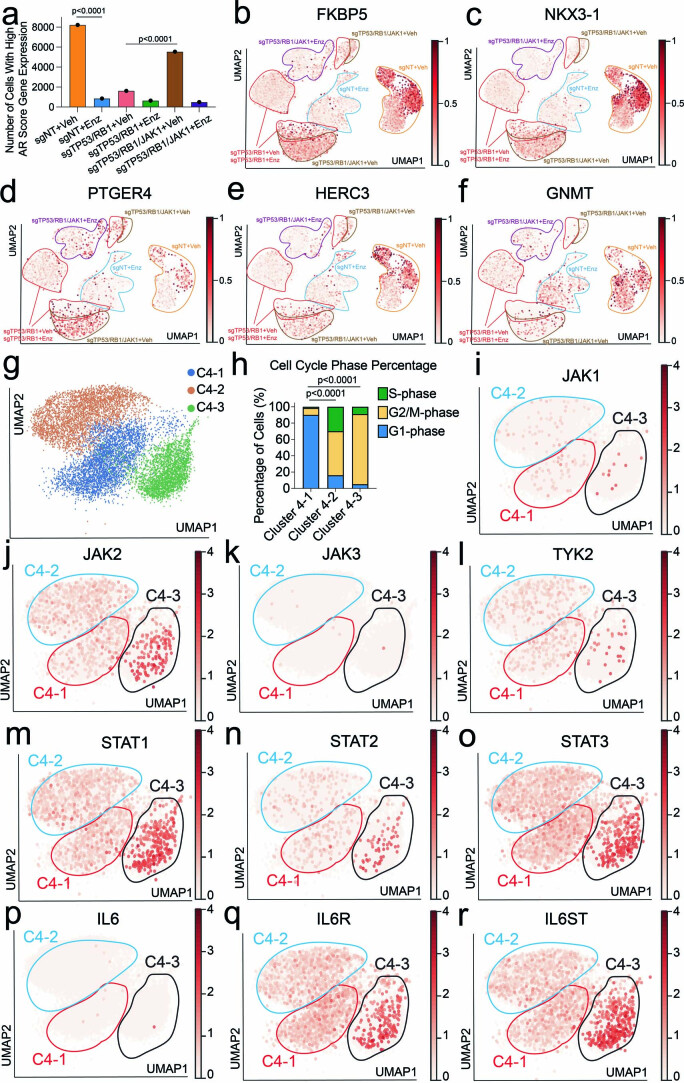
AR signaling partially restored in the subclones with TP53/RB1/JAK1-KD and vehicle treatment. a, Bar plot presents the number of single cells expressing high level (expression level in the top 20% of all single cells of all samples) of AR targeted genes (partial AR Score genes as shown in Supplemental Table). p-values are calculated with two-sided Chi-square test with Yates correction and annotated in figure. (Veh n = 14268 cells, Enz n = 15149 cells), sgTP53/RB1 (Veh n = 12267 cells, Enz n = 9850 cells), sgTP53/RB1/JAK1 (Veh n = 25200 cells, Enz n = 11096 cells). b-f, UMAP plot of single cell transcriptomic profiles colored by expression of selected AR target genes (z-score, AR Score genes) for each cell (dot). LNCaP/AR cells were transduced with Cas9 and annotated CRISPR guide RNAs and treated with vehicle or Enz for 5 days. Fields of different sample groups are labeled with different color. g, UMAP plot of single cells in cluster 4, colored by unsupervised clustering of 3 sub-clusters. h, Bar plot presents the percentage distribution of each single cell in different cell cycle phases from subcluster 4-1, 4-2 and 4-3. n of cells in each cluster: C4-1 n = 3680 cells, C4-2 n = 3459 cells, C4-3 n = 2886 cells. p-values are calculated with two-sided Fisher’s Exact Test. i-r, UMAP plot of single cell transcriptomic profiles colored by expression of canonical JAK–STAT target genes (z-score) for each cell (dot) of LNCaP/AR cells in Cluster 4. For panel i-r, distribution area of subcluster 4-1, 4-2, 4-3 are labeled with red, blue, and black. For all panels, color density of each cell is scaled by the color bar and p-values were annotated in figures. Source data

To examine the role of JAK–STAT in heterogeneous cell subpopulations, we performed scRNA-seq and transcriptomic analysis using the series of LNCaP/AR cell lines treated with Enz or vehicle. As expected, clustering of the sequenced cells was primarily driven by genetic and treatment perturbations (Fig. 6a–c). Interestingly, the majority of both the sgNT and sgTP53/RB1/JAK1 cells were clearly separated by different treatments (Fig. 6a,c), while sgTP53/RB1 cells did not display a similar separation (Fig. 6b). These data support the observation that a majority of the sgTP53/RB1 cells exhibit Enz resistance. Because AR antagonists can promote PCa cell cycle arrest^57^, we performed cell cycle prediction analysis and observed a dramatically increased cell cycle arrest occurring in the sgNT cells treated with Enz (Fig. 6a,d). By contrast, Enz treatment did not increase the population of sgTP53/RB1 cells in G1 phase, suggesting that the majority of sgTP53/RB1 cells are resistant to Enz (Fig. 6b,d). Remarkably, *JAK1* KO substantially increased the percentage of cells entering G1 after Enz treatment compared to that observed in the vehicle-treated group (Fig. 6c,d). These data validate the specific role of JAK–STAT in mediating AR-targeted therapy resistance. To further assess the dynamics of resistance, we investigated whether AR signaling was restored in resistant subclones. Not surprisingly, the sgNT + vehicle group consisted of the greatest number of cells expressing canonical AR score genes (Supplementary Table 7), and inhibition of their expression was subsequently verified after Enz exposure (Extended Data Fig. 10a–f). By contrast, both sgTP53/RB1 vehicle and sgTP53/RB1 Enz groups lacked expression of AR genes, supporting the emergence of AR-independent transcriptional programs (Extended Data Fig. 10a–f). The expression of AR targets was largely reestablished in many cells belonging to the sgTP53/RB1/JAK1 + vehicle group (two-thirds of AR score genes; Supplementary Table 7) compared to that observed in the sgTP53/RB1 + vehicle group (Extended Data Fig. 10a–f). These data suggest a partial restoration of AR signaling and AR dependency among the sgTP53/RB1/JAK1 cells.

To characterize lineage-specific tumor heterogeneity in resistant PCa cells, we performed unsupervised graph clustering (uniform manifold approximation and projection (UMAP))^58^ and identified six distinct cell subsets labeled as clusters 0–5, with further partitioning to 13 subclusters (Fig. 6e,f). Consistent with transcriptomic changes caused by TP53/RB1/JAK1 modification, five of the six clusters (clusters 0–4) predominantly overlapped with the clusters identified by genetic and treatment perturbations (Fig. 6g), while cluster 5 is a mixture of a small fraction of cells from five groups (Fig. 6e–g). To examine the cell proliferation state of these clusters, we overlapped the transcriptomic-based clustering with cell cycle prediction (Fig. 6h). Interestingly, cells within clusters 0, 1, 3 and 5 remain proliferative (termed the ‘winner’ clusters; Fig. 6i), whereas cluster 2 contains a much higher percentage of cells in cell cycle arrest (termed the ‘loser’ cluster; Fig. 6i). Lastly, cells within cluster 4 express elevated levels of cell cycle phase heterogeneity (Fig. 6h), a finding that will be expounded on later.

### JAK–STAT signaling is required for stem-like and multilineage clones

**Fig. 7:**
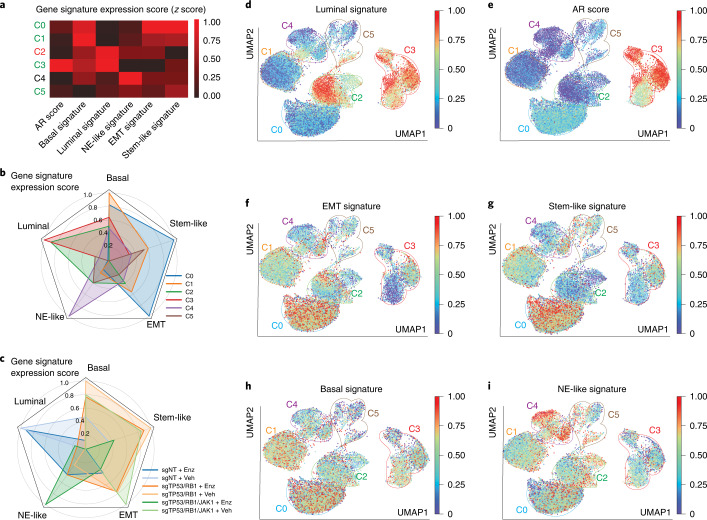
JAK–STAT is required for stem-like and multilineage subclones. **a**, Heat map representing the lineage scores of canonical lineage marker gene signatures in cell clusters. Winner clusters (without increased cell cycle arrest) are highlighted in green, and the loser cluster (with increased cell cycle arrest) is highlighted in red. **b**, Radar plot representing the lineage scores and distribution of different cell clusters. **c**, Radar plot representing the lineage scores and distribution of different samples. In **a**–**c**, lineage scores were scaled from 0 to 1 across all clusters. **d**, UMAP plot of single-cell transcriptomic profiles colored by luminal gene signature score (*z* score) for each cell (dot). **e**, UMAP plot of single-cell transcriptomic profiles colored by AR gene signature score (*z* score) for each cell (dot). **f**, UMAP plot of single-cell transcriptomic profiles colored by EMT gene signature score (*z* score) for each cell (dot). **g**, UMAP plot of single-cell transcriptomic profiles colored by stem cell-like gene signature score (*z* score) for each cell (dot). **h**, UMAP plot of single-cell transcriptomic profiles colored by basal gene signature score (*z* score) for each cell (dot). **i**, UMAP plot of single-cell transcriptomic profiles colored by NE-like gene signature score (*z* score) for each cell (dot). In **d**–**i**, distribution areas of each cluster are labeled in color circles. The color density of each cell is scaled by the color bar. For all data, the numbers (*n*) of cells in each sample and cluster are the same as in Fig. 6, and lineage scores were scaled from 0 to 1 across all cells.

We next probed the well-established AR score and five lineage-specific gene signatures (Supplementary Table 7)^5,24,59–61^and analyzed the expression of genes (*z* score) comprising these signatures across all clusters and samples (Fig. 7a–c). In congruence with the luminal epithelial cell lineage of LNCaP/AR cells, cluster 2 and cluster 3, which consist of cells originating from the sgNT groups, represent the two clusters expressing the highest level of luminal genes (Fig. 7a–d). Most of cluster 2 cells, while retaining their luminal lineage, displayed loss of AR signaling gene expression and entered cell cycle arrest following Enz administration (Fig. 7a–e). Notably, the most substantial proportions of clusters 0 and 1, consisting primarily of cells originating from the sgTP53/RB1 groups, expressed the lowest levels of the luminal gene signature and relatively high levels of non-luminal and multilineage gene signatures (Fig. 7a–i). Surprisingly, clusters 0 and 1 also contained a proportion of cells from the sgTP53/RB1/JAK1 + vehicle group, which maintained non-luminal programs (Fig. 7b–i), supporting the hypothesis that JAK–STAT inhibition does not impair the survival of those subclones in the absence of Enz (Figs. 6i and 7b,c). However, Enz dramatically diminished the survival of sgTP53/RB1/JAK1 subclones and the expression of stem-like and multilineage programs, suggesting that JAK–STAT inactivation restored AR dependency and impaired lineage plasticity (Fig. 7b,c). This hypothesis is further supported by restored AR signaling in sgTP53/RB1/JAK1 subclones (Extended Data Fig. 10a–f). Interestingly, *JAK1* KO did not substantially impair the resistance of subclones only expressing an NE-like lineage program (Fig. 7b,c,i), indicating that JAK–STAT signaling is specifically required for the transition to a stem-like and multilineage state rather than the transition to an exclusive NE-like state.

**Fig. 8:**
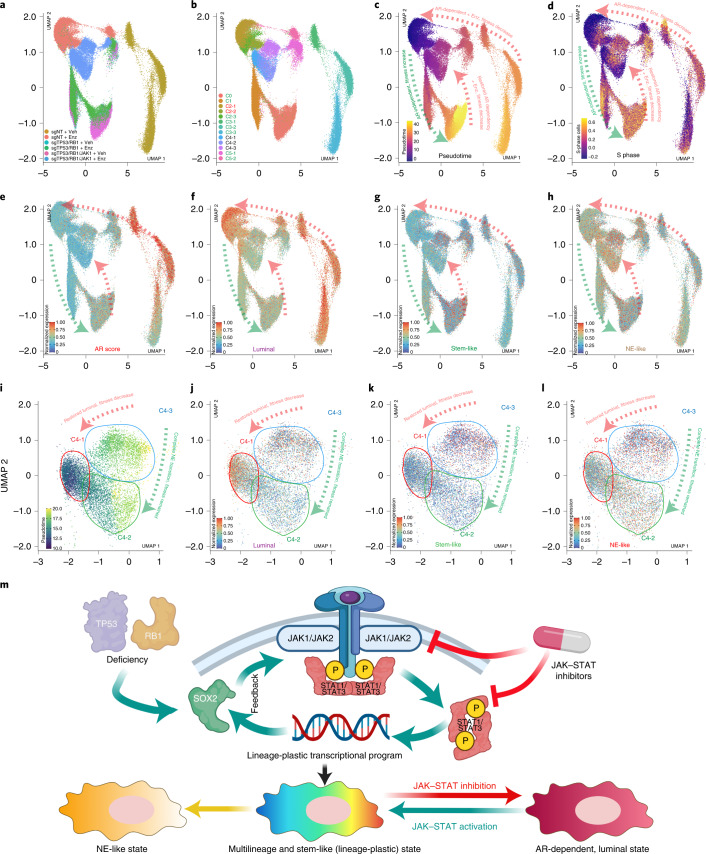
Dynamics of lineage plasticity driven by ectopic JAK–STAT activation. **a**, UMAP plots represent the reconstructive trajectory of single cells in each of the samples. **b**, UMAP plots represent the reconstructive trajectory of single cells in each of the subclusters. **c**, UMAP plots represent the pseudotime reconstructive trajectory of single cells. Color intensity represents the pseudotime estimation of each single cell. Arrows and the dotted line represent the direction of pseudotime flow. **d**, UMAP plots represent the S phase score per cell in each single cell within the pseudotime reconstructive trajectory. **e**–**h**, UMAP plots represent the AR signaling and lineage scores per cell in each single cell within the pseudotime reconstructive trajectory. **i**, UMAP plots represent the pseudotime reconstructive trajectory of single cells of cluster 4. Color intensity represents the pseudotime estimation of each single cell. Arrows and the dotted line represent the direction of pseudotime flow. **j**–**l**, UMAP plots represent lineage scores per cell in each single cell within the pseudotime reconstructive trajectory of single cells of cluster 4. **m**, Schematic figure illustrating that SOX2 ectopically activates JAK–STAT signaling, which enables the transition of mCRPC to a stem-like and multilineage state. Figure created with BioRender.com. The numbers (*n*) of cells in each sample and cluster are the same as in Fig. 6.

To decipher the dynamics of lineage plasticity, we performed pseudotime reconstructing trajectory analysis (Fig. 8a–c). We started with the transcriptional landscape of the only loser cluster, cluster 2, and tracked the changes of cell proliferation and lineage states. The increased pseudotime correlated with cell fitness, as reflected by an increased percentage of cells with active cell cycle and proliferation (Fig. 8c,d). Because clusters 2 and 3 predominantly contain wild-type sgNT cells (Fig. 7e,f), Enz treatment caused a substantial decrease of both cell fitness and pseudotime of the luminal and AR-dependent cells in those two clusters (Fig. 8c,d). Genetic perturbation of *TP53*/*RB1* KO (clusters 0 and 1) led to the transition to a multilineage and stem-like state, which confers an increase in cell fitness and pseudotime (Fig. 8a–d,f–h). Interestingly, *JAK1* KO did not immediately impair fitness nor reduce pseudotime of multilineage subclones but rather restored AR signaling in those cells (Fig. 8e). Indeed, Enz substantially impaired the fitness of those *JAK1* KO subclones and led to a decrease in pseudotime (Fig. 8c,d), supporting the hypothesis that JAK–STAT inhibition restored AR dependency of those cells. Notably, the subclones only expressing NE-like lineage maintained both high fitness and pseudotime (Fig. 8h), suggesting that JAK–STAT signaling is inessential for those subclones.

We continued to explore the lineage heterogeneity of the subclusters of cluster 4 (Fig. 6f and Extended Data Fig. 10g), which contain cells originating from the sgTP53/RB1/JAK1 + Enz group (Fig. 6e,g). The three subclusters of cluster 4 expressed diverse levels of the JAK–STAT signaling genes (Extended Data Fig. 10i–r), presumably because JAK–STAT signaling was not fully deactivated in a proportion of *JAK1*-KO cells due to compensatory signaling driven by JAK2 (Extended Data Fig. 10j). Cluster 4-3 contained the ‘outlier’ cells, which partially maintain JAK–STAT signaling, likely driven by JAK2 (Figs. 6f and 8i and Extended Data Fig. 10j). Remarkably, the cells within cluster 4-3 maintained expression of multilineage programs as well as the highest level of cell fitness, regardless of treatment conditions (Fig. 8i–k and Extended Data Fig. 10h). The other two subclusters of cluster 4 demonstrated two contrasting fates following deactivation of JAK–STAT signaling. Cluster 4-1 cells, which lose the multilineage and stem-like programs, restored the exclusive expression of the luminal program (Fig. 8j). Thus, cells of this subcluster were highly responsive to Enz (Extended Data Fig. 10h), which caused a substantial diminishment in cell fitness (Fig. 8i). By contrast, the cells of cluster 4-2, which exclusively express NE-like lineage programs, maintained cell fitness even in the absence of JAK–STAT signaling (Fig. 8l and Extended Data Fig. 10h), supporting the hypothesis that JAK–STAT signaling is not required for the cells fully differentiated to an NE-like state. The juxtaposition between different subclusters of cluster 4 further supports the crucial role of ectopic JAK–STAT signaling in maintaining AR therapy resistance of stem-like and multilineage subclones rather than subclones exclusively expressing an NE-like lineage (Fig. 8m).

## Discussion

Emerging evidence demonstrates that lineage plasticity represents an important mechanism for conferring targeted therapy resistance in various cancers, particularly prominent in cancers where the molecular target of therapies are lineage-specific survival factors^2^. In the case of PCa, however, it is not fully understood whether differentiated luminal tumor cells acquire lineage plasticity through reverting back (dedifferentiating) to a multilineage, stem cell-like state and then redifferentiating to alternative lineages or through direct transdifferentiation to a distinctively new lineage. Another intriguing feature of lineage plasticity-driven resistance is the elevated levels of intratumoral heterogeneity^62^, which increases the difficulty to dissect the molecular mediators required either for multilineage plasticity or for a specific lineage program, such as NE-like lineage, through the analysis of bulk cell population. Thus, the identification of lineage heterogeneous TP53/RB1-deficient tumor cell subpopulations through single-cell transcriptomic analyses illuminates these once hidden details and represents a major insight into this work. Here, we showed that a vast majority of the TP53/RB1-deficient tumor cells acquire lineage plasticity by transitioning to a stem-like, multilineage and AR-independent state. Importantly, our data acquired from the Dox-inducible model, as well as the pseudotime trajectory analysis, suggested that ectopic JAK–STAT activation is required for AR therapy resistance of those stem-like cells expressing multilineage programs rather than cells having undergone complete transition to an exclusive NE-like lineage.

Various genetic and transcriptional aberrations have been connected to lineage plasticity in PCa^3,6–9,12,13,18,61^. Interestingly, many of those cases involve the ‘hijacking’ of stem-like, pluripotency or epigenetic regulation programs^4,5,7,8,10,63^. Although the role of the JAK–STAT signaling pathway in regulating cell fate decision, stem cell self-renewal and multilineage differentiation has been well documented^27,28^, its potential function in mediating lineage plasticity remains largely unclear. Furthermore, the consequence of constitutive activation of STAT proteins in tumorigenesis is highly context specific^29,30^. Our results revealed that SOX2 regulates the ectopic induction of JAK–STAT signaling in a positive feedback and cell-autonomous fashion. Consequently, JAK–STAT activation, in a JAK1/JAK2- and STAT1/STAT3-dependent manner, is required for the transition to a stem-like, multilineage and EMT state but not for the tumor cells that have completely redifferentiated to an NE-like lineage. The results of combinatorial KO and pharmaceutical inhibition of various JAK–STAT signaling proteins suggests that those proteins, specifically JAK1/JAK2 and STAT1/STAT3, may function in a collaborative and compensatory network to confer lineage plasticity. Our results also reveal that ectopic JAK–STAT expression enables an EMT lineage program that promotes a metastatic phenotype.

Despite the clinical success of AR-targeted therapies, resistance to these treatments universally develops and largely impairs the clinical outcome of individuals with mCRPC. Although lineage plasticity has been suggested as a substantial mechanism conferring resistance, effective therapeutic approaches targeting lineage plasticity are still not available^2^. Here, we demonstrated that various pharmaceutical inhibitors targeting different JAK and STAT proteins have combinatorial effects when administered with Enz. These results may provide a rationale for future clinical trials designed to target JAK–STAT signaling for overcoming lineage plasticity-driven AR-targeted therapy resistance. Finally, it is crucial to place our model of how JAK–STAT signaling promotes lineage plasticity-driven resistance within the context of TP53 and RB1 deficiency. Although the connection between JAK–STAT activation and TP53/RB1 alterations are well documented in various cancers^64,65^, an inverse correlation between wild-type TP53 and JAK–STAT activation is also widely reported^66,67^. These results are consistent with our finding that the inactivation of JAK–STAT signaling impairs proliferation of resistant cells with TP53/RB1 deficiency while not affecting cells with intact TP53/RB1. Therefore, it is critical to consider the genomic state of *TP53*/*RB1* when correlating JAK–STAT activation with the clinical outcome of AR therapy responses, as JAK–STAT activation in individuals with wild-type TP53/RB1 may not be a consequence of lineage hijacking but rather a cytokine-induced immune response.

## Methods

### Ethics statement

All animals were housed under humidity- and temperature-controlled conditions with a 12-h light/12-h dark cycle in the pathogen-free facilities at UT Southwestern Medical Center by the Animal Resource Center and were monitored closely to minimize discomfort, distress, pain or injury throughout the course of the in vivo experiments. Animals were removed from the study and killed if any signs of pain and distress were detected or if the tumor volume reached 2,000 mm^3^. The maximal tumor size was not exceeded in all reported studies. All procedures were performed in accordance with the recommendations of the Panel on Euthanasia of the American Veterinary Medical Association, and the animal protocol was approved by Institutional Animal Care and Use Committee of UT Southwestern Medical Center (protocol 2019-102493). Male C.B-*lgh*-*1*^*b*^/lcrTac-*Prkdc*^scid^ SCID mice were obtained from Taconic Biosciences.

### Cell lines and organoid culture

Information and requests for resources and reagents should be directed to and will be fulfilled by the corresponding author. All cell lines, plasmids and other reagents generated in this study are available from the corresponding author with a completed materials transfer agreement if there is potential for commercial application. Parental LNCaP/AR and CWR22Pc PCa cell lines were obtained from the laboratory of C. Sawyers at Memorial Sloan Kettering Cancer Center (MSKCC)^5^, and Du145 (HTB-81) and PC3 (CRL-1435) cell lines were purchased from ATCC. LNCaP/AR, CWR22Pc and PC3 cells were cultured in RPMI 1640 medium supplemented with 10% fetal bovine serum (FBS), 1% l-glutamine, 1% penicillin–streptomycin, 1% HEPES and 1% sodium pyruvate. DU145 cells were cultured in DMEM high-glucose medium supplemented with 10% FBS, 1% l-glutamine and 1% penicillin–streptomycin. LNCaP/AR, PC3 and DU145 cells were passaged at a 1:6 ratio every 3–5 d, and CWR22Pc cells were passaged at a 1:3 ratio every 3–5 d. When treated with 10 µM Enz and/or 5 µM Filg, LNCaP/AR cells were cultured in RPMI 1640 medium supplemented with 10% charcoal-stripped serum (CSS medium). All cell cultures were assessed for mycoplasma monthly via a MycoAlert Plus Mycoplasma Detection kit (Lonza, LT07-710), and all results were negative. Cell line identification was validated through human short tandem repeat profiling cell authentication and was compared to ATCC cell line profiles every year. *Trp53*^loxP/loxP^*Rb1*^loxP/loxP^ mouse organoids were generated from *Trp53*^loxP/loxP^*Rb1*^loxP/loxP^ mice^5^. Human organoids were obtained from the laboratory of Y. Chen at MSKCC^54,55^. Organoids were cultured in 3D Matrigel according to the previously described protocol^53,68^. Organoids were split at a 1:10 (mouse) or 1:3 (human) ratio every 5 d.

### CRISPR and shRNA

Lentiviral-based constructs were used for CRISPR-based KO or shRNA-based knockdown of all genes modified in the manuscript^12^. LNCaP/AR cells were seeded at 400,000 cells per well in 2 ml of medium in six-well plates. Medium was replaced with medium containing 50% virus, 50% fresh culture medium and 5 μg ml^–1^ polybrene the next day. The lentiviral virus-containing medium was replaced with normal culture medium after 24 h. Cells were selected with 2 μg ml^–1^ puromycin for 4 d or 5 μg ml^–1^ blasticidin for 5 d. For cells with double colors, transduced cells were further sorted with a flow cytometer. Human DYKDDDDK (Flag)-tagged SOX2 expression lentivirus (337402) was purchased from Qiagen and used for direct cell transduction, following the manufacturer’s instruction. The All-In-One lentiCRISPRv2 (Addgene plasmid 52961), LentiCRISPRv2GFP (Addgene plasmid 82416), LentiCRISPRv2-mCherry (Addgene plasmid 99154), pLKO5.sgRNA.EFS.RFP (Addgene plasmid 57823), pLKO5.sgRNA.EFS.GFP and lentiCas9-Blast (Addgene plasmid 52962) plasmids were used to generate the CRISPR and guide RNAs. Guide RNA constructs with an empty space holder served as the sgNT control. Guide RNAs were designed using the Benchling guide RNA designing tool (https://benchling.com). shRNA constructs SGEP (pRRL-GFP-miRE-PGK-PuroR) and LT3GEPIR (pRRL-TRE3G-GFP-miRE-PGK-PuroR-IRES-rtTA3) were originally obtained from the laboratory of J. Zuber at the Research Institute of Molecular Pathology. Sequences of sgRNAs and shRNAs are listed in Supplementary Table 8.

### In vivo xenograft experiment

All animal experiments were performed in compliance with the guidelines of the Animal Resource Center of UT Southwestern. LNCaP/AR in vivo xenograft experiments were performed by subcutaneous injection of 2 × 10^6^ cells, which were suspended in 100 μl in 50% Matrigel and 50% growth medium, into the flanks of castrated male C.B-Igh-1b/Icr Tac-*Prkdc*^scid^ SCID mice on both sides. For the experiment depicted in Fig. 1e, daily gavage treatment with 10 mg kg^–1^ Enz or vehicle (1% carboxymethyl cellulose, 0.1% Tween 80 and 5% DMSO) was started 1 d after the injection. Tumor size was measured weekly by digital caliper because tumors were noticeable. For experiments depicted in Fig. 4b, 10 mg kg^–1^ Enz (daily) and/or 20 mg kg^–1^ Filg (twice daily) were given after 3 weeks of Enz-only administration when tumors averaged around 200 mm^3^ in size.

### Cell dose–response curve, growth, viability and fluorescence-activated cell sorting-based competition assays

For the viability assay and dose–response curve, 4,000 LNCaP/AR cells were seeded in each well of a 96-well plate and treated with the annotated treatments for 8 d before conducting the assay. Cell viability was then measured by CellTiter-Glo luminescent cell viability assay (Promega, 7570) according to manufacturer’s protocol by using a SpectraMax iD3 automatic plate reader^12^. For the cell growth assay, LNCaP/AR (10,000 cells per well) or CWR22Pc (50,000 cells per well) cells were seeded in a 24-well plate in FBS medium (CWR22Pc) or CSS medium (LNCaP/AR) and treated with Enz (10 μM for LNCaP/AR and 1 μM for CWR22Pc) or vehicle (DMSO) for 7 d (LNCaP/AR) or 4 d (CWR22Pc). Cell numbers were counted by a Countess II FL automatic cell counter (Invitrogen). For the organoid growth assay, 2,000 mouse organoid cells were seeded in 3D Matrigel (per 50-µl sphere) in murine organoid medium with Enz and/or Filg for 6 d. Matrigel was washed away with cell recovery medium (Corning, 354253), and organoids were separated into single-cell suspensions by treatment with trypsin. Cell numbers were counted, and the relative cell growth (treatments/vehicle) was calculated. For fluorescence-activated cell sorting (FACS)-based competition assays, the competition cell mixture of sgTP53/RB1-RFP cells and sgNT-GFP cells was treated with Enz (10 μM), and the percentages of RFP-positive cells were measured on day 0, day 4 and day 8 by FACS. LNCaP/AR cells were first gated based on SSC-H/FSC-A→FSC-H before measuring the RFP/GFP signals. Relative cell number fold change was calculated and normalized to the vehicle-treated group, as previously described^12^. Attune Nxt (version 4.2.1627.1) and FlowJo (version 10.8.0) were used for FACS data analysis.

### Chemicals

Enz was purchased from the Organic Synthesis Core Facility at MSKCC. Filg and ruxolitinib are commercially available from MedChem Express (Filg, HY-18300; ruxolitinib, HY-50856). Fludarabine and niclosamide are commercially available from Selleck Chemicals (fludarabine, S1491; niclosamide, S3030).

### Migration, invasion and prostasphere assays

For the migration assay, 20,000 LNCaP/AR cells were resuspended in serum-free RPMI and seeded in the upper transwell insert (Corning, 353097)^69^. RPMI with 10% serum was added to the lower chamber as a chemoattractant. After 60 h of incubation, cells that migrated to the lower side of the transwell insert were fixed with paraformaldehyde and stained with 1% crystal violet. Images were acquired on a Leica DMi8 inverted microscope. Nine representative images of each group were used to quantify the migrated cell numbers. For the invasion assay, inserts were coated with extracellular matrix gel (Corning, 354234) before plating. Stock Matrigel (10 mg ml^–1^) was thawed overnight at 4 °C and diluted in serum-free RPMI to 30 μg per insert. Each insert was then coated with 100 μl of diluted Matrigel and incubated for 1 h at 37 °C with 5% CO_2_. Cells were then seeded at the same density as the migration assay. Cells were fixed and stained with 1% crystal violet after 60 h, and the invading cell numbers were quantified by using ImageJ, as in the migration assay. The prostasphere assay method was adapted from previous reports^70^. Two hundred cells were seeded into each well of a 96-well ultralow attachment plate. For each condition, three wells were prepared for statistical analysis. Prostaspheres were imaged at one picture/well and quantified 7 d after seeding. Culture medium used in this experiment was basic organoid medium supplemented with 20 ng ml^–1^ epidermal growth factor and 10 ng ml^–1^ basic fibroblast growth factor. All images were quantified by using ImageJ (version 2.0.0).

### PDE and PDO experiments

PDE models were established in the Raj laboratory, as previously described^48,49^. PDEs of ~1 mm^3^ were cultured in a sponge with RPMI 1640 medium with 10% FBS, 1× penicillin–streptomycin (PS) solution, 0.01 mg ml^–1^ hydrocortisone and 0.01 mg ml^–1^ insulin. PDEs were treated with 10 µM Enz or DMSO for 24 h before RNAs were collected. PDO models were established in the Chen laboratory^53–55^. PDOs were cultured in 3D Matrigel with typical human organoid medium according to the previously published protocol^53^. Organoids were split at a 1:3 ratio every 7 d by using trypsin or a sterile glass pipette. When treated with 1 μM Enz and/or 5 μM Filg, these organoids were cultured in typical human organoid medium supplemented with drugs.

### Gene and protein expression detection by qPCR and western blotting

Total RNA from cells was extracted by using Trizol (Ambion, 15596018), and cDNA was made using SuperScript IV VILO Master Mix with ezDNase enzyme (Thermo Fisher, 11766500) with 200 ng µl^–1^ RNA template. cDNA was amplified with 2× PowerUp SYBR Green Master Mix (Thermo Fisher, A25778). For western blotting, proteins were extracted from cell lysates using RIPA buffer and measured with a Pierce BCA Protein Assay kit (23225). Protein lysates were boiled at 95 °C for 5 min and run on NuPAGE 4–12% Bis-Tris gels (Invitrogen, NP0323). Transfer was conducted for 1 h at 100 V at 4 °C. Membranes were then blocked for 15 min in 5% non-fat milk before incubation with primary antibody and washed with 1× TBST (10× stock from Teknova, T9511). The following antibodies were used for western blotting: JAK1 (Cell Signaling Technology, 3332S), STAT1 (Cell Signaling Technology, 9172S), p-STAT1(58D6) (Cell Signaling Technology, 9167S), Rb1(4H1) (Cell Signaling Technology, 5230), P53(DO1) (Leica Biosystems, NCL-p53-DO1), actin(8H10D10) (Cell Signaling Technology, 3700). JAK2(C-10) (Santa Cruz, sc-390539), JAK3 (Cell Signaling Technology, 3775), STAT2(D9J7L) (Cell Signaling Technology, 72604), STAT3(D1B2J) (Cell Signaling Technology, 30835), vimentin(D21H3) (Cell Signaling Technology, 5741), ASCL1(EPR19840) (Abcam, ab211327), peroxidase AffiniPure goat anti-mouse IgG (H + L) (AB_10015289; Jackson ImmunoResearch, 115-035-003) and peroxidase AffiniPure goat anti-rabbit IgG (H + L) (AB_2313567; Jackson ImmunoResearch, 111-035-003). Dilutions of all primary antibodies were 1:1,000. Dilutions of all secondary antibodies were 1:5,000. Human and mouse qPCR primers are listed in Supplementary Table 9.

### IF and IHC staining

Tumors were collected and fixed in 4% paraformaldehyde and embedded in paraffin by the UT Southwestern Tissue Management Shared Resource core. Tumors were then sectioned at 5 µm, and hematoxylin and eosin and IHC staining were performed using standard protocols. Images were acquired on a Leica DMi8 microscope. Deidentified human PCa formalin-fixed paraffin-embedded slides were purchased from the UT Southwestern Tissue Management Shared Resource core, and IHC staining was performed using a standard protocol. Three-dimensional cultured organoids were washed with PBS and fixed in 4% paraformaldehyde for 90 min. Organoids were embedded in 2% agarose, and the agarose plug was sent to the UT Southwestern Tissue Management Shared Resource core for paraffin embedding. The paraffin-embedded organoids were then sectioned at 5 µm and stained following a standard IHC protocol. For 3D-cultured organoid IF staining^71^, organoids were fixed in 4% paraformaldehyde, permeabilized with 0.5% Triton X-100, blocked with 3D blocking buffer (2% bovine serum albumin, 0.1% Triton X-100 and 0.05% Tween 20) and incubated with Alexa Fluor 647-conjugated or unconjugated primary antibody at 37 °C for 48 h. Organoids were then washed in 3D IF buffer (PBS, 0.1% Triton X-100 and 0.05% Tween 20) and incubated with Alexa Fluor 647-conjugated secondary antibody and DAPI at 37 °C overnight. After washing with PBS, stained organoids were placed on slides, and images were acquired on a confocal microscope. For LNCaP/AR cell IF staining, cells were seeded on round glass coverslips. Twenty-four hours after seeding, cells were fixed with 4% paraformaldehyde and permeabilized with 0.5% Triton X-100. After blocking with 3% bovine serum albumin/PBS, cells were incubated with primary antibody at 4 °C overnight, and Alexa Fluor-labeled secondary antibodies were incubated with cells for 1 h at room temperature. DAPI was used for nuclei staining. Images were captured on a Zeiss LSM 700 confocal laser-scanning microscope. The following antibodies were used for IHC and IF staining: Jak1 (Cell Signaling Technology, 3332), Stat1 (Cell Signaling Technology, 14994), Stat3(D1B2J) (Cell Signaling Technology, 30835), Alexa Fluor 647 anti-cytokeratin 8(EP1628Y) (Abcam, ab192468), Alexa Fluor anti-cytokeratin 18(E431-1) (Abcam, ab194125, GR-200266-1), Alexa Fluor 647 anti-cytokeratin 5(EP1601Y) (Abcam, ab193895, GR-219431-2), Alexa Fluor 647 anti-cytokeratin 14(EP1612Y) (Abcam, ab192056), Nkx3.1(4H4) (Abcam, ab96482), PSA/KLK3(D6B1) (Cell Signaling Technology, 5365), NDRG1(D8G9) (Cell Signaling Technology, 9485), vimentin(D21H3) (Cell Signaling Technology, 5741), synaptophysin(D8F6H) (Cell Signaling Technology, 36406), Alexa Fluor 647-conjugated AffiniPure goat anti-mouse IgG (H + L) (AB_2338902; Jackson ImmunoResearch, 115-605-003), Alexa Fluor 647-conjugated AffiniPure goat anti-rabbit IgG (H + L) (AB_2338078; Jackson ImmunoResearch, 111-605-144), donkey anti-mouse IgG antibody (biotin-SP (long spacer)) (AB_2307438; Jackson ImmunoResearch, 715-065-150) and donkey anti-rabbit IgG antibody (biotin-SP (long spacer)) (AB_2340593; Jackson ImmunoResearch, 711-065-152). Dilutions of all primary antibodies were 1:200 except for JAK1 (1:100). Dilutions of all secondary antibodies were 1:1,000.

### ChIP–qPCR and SOX2 ChIP–seq

Cultured cells were cross-linked with 1% formaldehyde and quenched with 0.125 M glycine. Cells were then rinsed with cold 1× PBS twice and lysed in 1% SDS containing buffer supplemented with 1× protease and phosphatase inhibitors. Chromatin was sonicated to an average length of 500 base pairs and centrifuged at 14,000 r.p.m. to remove debris. One percent of the supernatant was saved as input, and the rest was added with ChIP-grade antibody overnight. Then, 20 µl of agarose/protein A or G beads was added and incubated for 4 h. Beads were washed with standard wash buffers (low-salt, high-salt and LiCl) and finally with TE. The immunoprecipitated chromatin was eluted in elution buffer and decross-linked by NaCl at 65 °C overnight. Proteins were then digested by proteinase K, and DNA was purified with a MinElute PCR purification kit (Qiagen, 28006) and eluted with 10 µl of water. Antibodies used included Sox2(D9B8N) (Cell Signaling Technology, 23064S), anti-histone H3 (acetyl K27) antibody ChIP-grade (Abcam, ab4729) and tri-methyl-histone H3 (Lys 27) (C36B11) rabbit monoclonal antibody (Cell Signaling Technology, 9733S). Dilutions of all antibodies were 1:100. Primer sequences are listed in Supplementary Table 9. SOX2 ChIP–seq data generated from the CWR-R1 and WA01 cells were described in Wet et al., and the SOX2 ChIP–seq data were downloaded from GSE166185 (ref. ^56^).

### Bulk RNA-seq preparation and analysis

LNCaP/AR cells were treated with Enz or vehicle for 6 d before total RNA was extracted using Trizol (Ambion, 15596018). RNA-seq libraries were prepared using the stranded Illumina TruSeq mRNA kit starting from 500 ng of total RNA with 10 cycles of PCR amplification. Barcoded RNA-seq libraries were run as paired-end, 50-nucleotide reads on an Illumina HiSeq 2500 and filtered by poly(A) selection. Alignment, quantification and differential analysis were performed using the QBRC_BulkRnaSeqDE pipeline (https://github.com/QBRC/QBRC_BulkRnaSeqDE). Briefly, alignment of reads to the human reference genome (GRCh38, https://www.ncbi.nlm.nih.gov/assembly/GCF_000001405.26) was done using STAR (v2.7.2b)^72^. FeatureCounts (v1.6.4)^73^ was used for gene counts, biotype counts and rRNA estimation. Differential expression analysis was performed using the R package DEseq2 (v1.26)^74^. Cutoff values of an absolute fold change greater than 2 and a false discovery rate of <0.1 were used to select for differentially expressed genes. GSEA was performed with the R package fgsea (v1.14.0) using the ‘KEGG’ and ‘Hallmark’ libraries from MsigDB.

### scRNA-seq preparation and analysis

LNCaP/AR cells were treated with Enz or vehicle for 5 d before the cells were collected. Single cells were sorted into 1.5-ml tubes, and the concentration was adjusted to 900–1,100 cells per μl. Cells were loaded between 10,000 and 17,000 cells per chip position using the Chromium Single Cell 5′ Library, Gel Bead & Multiplex kit and Chip kit (10x Genomics, V1 barcoding chemistry). Single-cell gene expression libraries were generated according to the manufacturer’s instructions, and single-cell expression sequencing was run on a NovaSeq 6000 (Novogene). The 10x scRNA-seq data were preprocessed using Cell Ranger software (5.0.0). We used the ‘mkfastq’, ‘count’ and ‘aggr’ commands to process the 10x scRNA-seq output into one cell by gene expression count matrix using default parameters. scRNA-seq data analysis was performed with the Scanpy (1.6.0) package in Python^75^. Genes expressed in fewer than three cells were removed from further analysis. Cells expressing less than 100 and more than 7,000 genes were also removed from further analysis. In addition, cells with a high (≥0.15) mitochondrial genome transcript ratio were removed. For downstream analysis, we used count per million (CPM) normalization to control for library size differences in cells and transformed those into log (CPM + 1) values. After normalization, we used the ‘pp.highly_variable_genes’ command in Scanpy to find highly variable genes across all cells using default parameters except for ‘min_mean = 0.01’. The data were then *z*-score normalized for each gene across all cells. We then used the ‘tl.pca (n_comps=50, use_highly_variable=True)’, the ‘pp.neighbors (n_pcs=25, n_neighbors=15)’ and the ‘tl.leiden (resolution = 0.75)’ commands in Scanpy to partition the single cells into six distance clusters. Briefly, these processes first identified 50 principal components in the data based on the previously found highly variable genes to reduce the dimensions in the original data and built a nearest neighbor graph based on the top 25 principal components, and a partition of the graph that maximizes modularity was found with the Leiden algorithm^76^. To evaluate the activity of lineage-specific transcriptional programs in those cells, we used a custom library of genes based on the well-established gene signatures for AR target genes (AR score) and NE, luminal, basal, stem-like and EMT lineages. The AR score gene signature was adapted from Hieronymus et al.^59^, and luminal, basal and NE gene signatures were defined by combining the signature genes from refs. ^5,24,60,61^. EMT and stem-like gene signatures were adapted from the signature genes of Dong et al.^24^ plus canonical lineage marker genes (Supplementary Table 7). The activation score was calculated based on the overall expression of genes in each gene list using the ‘tl.score_genes’ function of the Scanpy package. To evaluate and model lineage plasticity as a function of cell genotype, we performed trajectory analysis using the R package ‘Monocle 3’^77^. We provided the single-cell gene expression matrix containing only the highly variable genes defined as previously discussed as input and used principal-component analysis and UMAP during preprocessing steps. The trajectory was built using default parameters, with the root defined from the loser cluster. Human CRPC tumor biopsy single-cell data were downloaded from GSE137829 (ref. ^24^). We analyzed these data in a similar manner using the ‘scanpy’ Python package. Briefly, we kept only epithelial cells from the data, performed CPM normalization and a principal-component analysis and evaluated gene expression representing key signaling pathways and lineage-specific translational programs.

### Statistics and reproducibility

Statistical details of all experiments can be found in the respective figure legends. A two-sided *t*-test was used and adjusted for multiple comparisons (Welch’s correction) when applicable when two groups of independent datasets that fit normality and homoscedasticity were compared. When normality and homoscedasticity were not satisfied, a Mann–Whitney *U*-test (non-parametric Wilcoxon rank-sum test) was used when comparing gene expressions between two groups. For in vitro cell line studies, data distribution was assumed to be normal, but this was not formally tested. One-way or two-way analysis of variance (ANOVA) and Kruskal–Wallis non-parametric ANOVA were used as appropriate to compare more than two groups. The mean ± s.e.m. values were reported, and *P* values were calculated and adjusted for multiple comparisons (Bonferroni or Benjamini correction) when applicable. *P* values were calculated by non-linear regression with an extra sum-of-squares *F*-test for the dose–response curve. A two-sided Fisher’s exact test was used to compare the numbers of tumors with genomic alterations between different groups and the percentages of cell populations. A two-sided chi-squared test with Yates correction was used to compare the exact cell numbers of different clusters of single-cell subclones. For in vivo experiments, no statistical method was used to predetermine sample size, but our sample sizes were selected based on and are similar to those reported in previous studies^5,12,78–80^. No data were excluded from the analyses. For in vivo studies, the tumor xenografting, measurement and analysis were performed by different researchers to ensure that the studies were run in a blinded manner. Mice were randomized into each group. For in vitro studies, randomization and blinding of cell lines was not possible; however, all cell lines were treated identically without prior designation. Graph Pad Prism (V9.3.1) was used for data graphing and statistical analysis.

### Reporting summary

Further information on research design is available in the Nature Research Reporting Summary linked to this article.

## Supplementary information

Supplementary Information Supplementary Fig. 1. Gating strategy figure for FACS-based competition assay.

Reporting Summary

Supplementary Tables Supplementary Tables 1–9.

## Data Availability

All the described bulk and scRNA-seq data that support the findings of this study have been deposited in the Gene Expression Omnibus under the accession number GSE175975. The human CRPC tumor biopsy single-cell data were downloaded from and are available in the Gene Expression Omnibus under the accession number GSE137829 (ref. ^24^). Human genomic and transcriptomic data were derived from the TCGA research network and the SU2C cohort, which were queried using cbioportal (http://www.cbioportal.org/study/summary?id=prad_su2c_2019) and the Genomic Data Commons Data Portal (https://portal.gdc.cancer.gov/). SOX2 ChIP–seq data were downloaded from and are available under the accession number GSE166185 (ref. ^56^). RNA-seq reads were aligned to the human reference genome (GRCh38, https://www.ncbi.nlm.nih.gov/assembly/GCF_000001405.26/). Source data are provided with this paper. All other data supporting the findings of this study are available from the corresponding author on reasonable request.

## Source data

Source Data Fig. 1 Statistical source data for Fig. 1.
Source Data Fig. 2 Statistical source data for Fig. 2.
Source Data Fig. 3 Statistical source data for Fig. 3.
Source Data Fig. 4 Statistical source data for Fig. 4.
Source Data Fig. 5 Statistical source data for Fig. 5.
Source Data Fig. 6 Statistical source data for Fig. 6.
Source Data Extended Data Fig. 2 Statistical source data for Extended Data Fig. 2.
Source Data Extended Data Fig. 3 Statistical source data for Extended Data Fig. 3.
Source Data Extended Data Fig. 4 Statistical source data for Extended Data Fig. 4.
Source Data Extended Data Fig. 5 Statistical source data for Extended Data Fig. 5.
Source Data Extended Data Fig. 6 Statistical source data for Extended Data Fig. 6.
Source Data Extended Data Fig. 7 Statistical source data for Extended Data Fig. 7.
Source Data Extended Data Fig. 8 Statistical source data for Extended Data Fig. 8.
Source Data Extended Data Fig. 9 Statistical source data for Extended Data Fig. 9.
Source Data Extended Data Fig. 10 Statistical source data for Extended Data Fig. 10.
Source Data Extended Data Fig. 2 Unprocessed blot for Extended Data Fig. 2.
Source Data Extended Data Fig. 3. Unprocessed blot for Extended Data Fig. 3.
Source Data Extended Data Fig. 4 Unprocessed blot for Extended Data Fig. 4.

## Code availability

All analyses in this work were done using open-source software. Bulk RNA-seq analysis was done using the QBRC Bulk RNA-seq pipeline (https://github.com/QBRC/QBRC_BulkRnaSeqDE). Briefly, reads were aligned to a reference (GRCh38) with ‘STAR’ (v2.7.2b). Gene counts were quantified with ‘FeatureCounts’ (v1.6.4). Differential gene expression analysis was performed using the R package ‘DEseq2’ (v1.26). GSEA statistical analysis was performed with the R package ‘fgsea’ (v1.14.0). Demultiplexing, alignment and read counting of the scRNA-seq data were performed using the 10x Genomics Cell Ranger 5.0.0 software. scRNA-seq data analysis was performed with the ‘Scanpy’ (v1.6.0) package in Python. Custom codes for the analysis in the paper have been deposited to GitHub and can be accessed at 10.5281/zenodo.6888969.
