# Author Correction: Ectopic JAK–STAT activation enables the transition to a stem-like and multilineage state conferring AR-targeted therapy resistance

**DOI:** 10.1038/s43018-022-00458-y

**Published:** 2022-10-14

**Authors:** Su Deng, Choushi Wang, Yunguan Wang, Yaru Xu, Xiaoling Li, Nickolas A. Johnson, Atreyi Mukherji, U-Ging Lo, Lingfan Xu, Julisa Gonzalez, Lauren A. Metang, Jianfeng Ye, Carla Rodriguez Tirado, Kathia Rodarte, Yinglu Zhou, Zhiqun Xie, Carlos Arana, Valli Annamalai, Xihui Liu, Donald J. Vander Griend, Douglas Strand, Jer-Tsong Hsieh, Bo Li, Ganesh Raj, Tao Wang, Ping Mu

**Affiliations:** 1 Department of Molecular Biology, UT Southwestern Medical Center, Dallas, TX USA; 2 Quantitative Biomedical Research Center, Department of Population and Data Sciences, UT Southwestern Medical Center, Dallas, TX USA; 3 Department of Urology, UT Southwestern Medical Center, Dallas, TX USA; 4 Department of Pathology, Duke University School of Medicine, Durham, NC USA; 5 Lyda Hill Department of Bioinformatics, UT Southwestern Medical Center, Dallas, TX USA; 6 Department of Neuroscience, UT Southwestern Medical Center, Dallas, TX USA; 7 Wakeland Genomics Core, UT Southwestern Medical Center, Dallas, TX USA; 8 Department of Pathology, The University of Illinois at Chicago, Chicago, IL USA; 9 Hamon Center for Regenerative Science and Medicine, UT Southwestern Medical Center, Dallas, TX USA; 10 Harold C. Simmons Comprehensive Cancer Center, UT Southwestern Medical Center, Dallas, TX USA

**Keywords:** Prostate cancer, Cancer therapeutic resistance, Targeted therapies, Tumour heterogeneity, Cancer

Correction to: *Nature Cancer* 10.1038/s43018-022-00431-9, published online 5 September 2022.

In the version of this article initially published, the affiliation listed for Jer-Tsong Hsieh was incorrect. The correct affiliation (Department of Urology, UT Southwestern Medical Center, Dallas, TX, USA) has now been inserted in the HTML and PDF versions of the article.

