# Supplementary material for: Ectopic JAK–STAT activation enables the transition to a stem-like and multilineage state conferring AR-targeted therapy resistance
Source: Nat Cancer. 2022 Sep 5;3(9):1071–87. doi: 10.1038/s43018-022-00431-9 (PMC9499870; doi:10.1038/s43018-022-00431-9)

nature portfolio

https://doi.org/10.1038/s43018-022-00431-9

Supplementary information

Ectopic JAK–STAT activation enables the transition to a stem-like and multilineage state conferring AR-targeted therapy resistance

In the format provided by the authors and unedited

Supplementary Figure 1. Flow cytometry gating strategy for FACS-based competition assay:

Gating strategy for FACS-based competition assay, where the percentage of RFP+ (sgTP53/RB1) and GFP+ (sgNT) cells were measured. LNCaP/AR cells were first gated based on SSC-A/FSC-A/FSC-H before measuring the RFP/GFP signals.

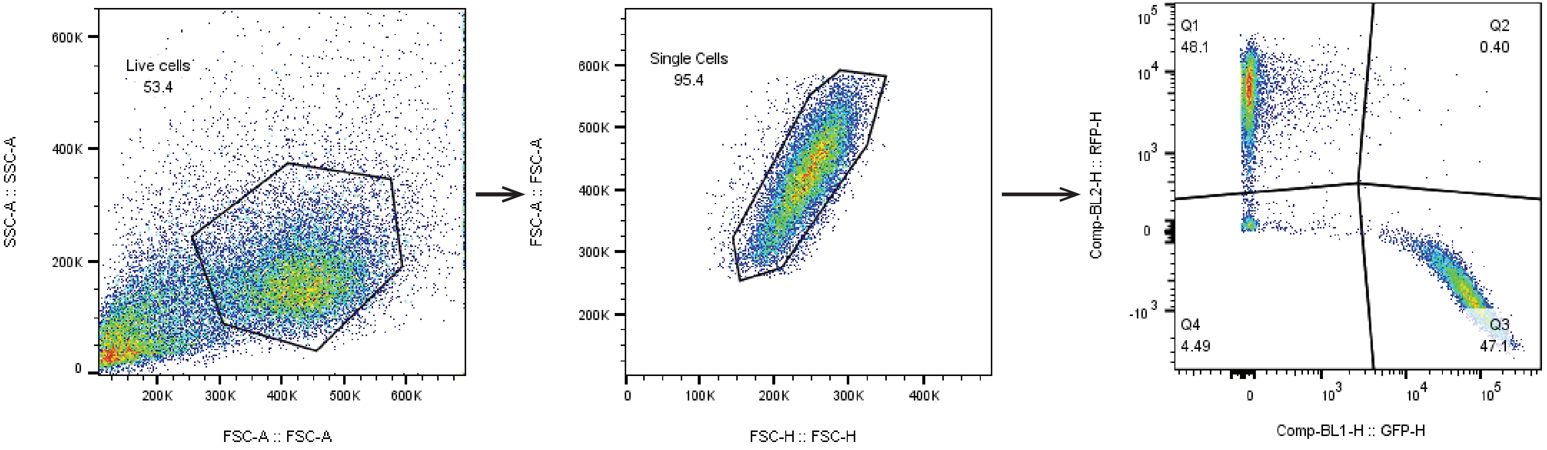

Supplement: Supplementary file 1 — Supplementary Fig. 1. Gating strategy figure for FACS-based competition assay. [file 43018_2022_431_MOESM1_ESM.pdf]
