# Supplementary material for: Ectopic JAK–STAT activation enables the transition to a stem-like and multilineage state conferring AR-targeted therapy resistance
Source: Nat Cancer. 2022 Sep 5;3(9):1071–87. doi: 10.1038/s43018-022-00431-9 (PMC9499870; doi:10.1038/s43018-022-00431-9)
Supplement: Supplementary file 2 — Reporting Summary [file 43018_2022_431_MOESM2_ESM.pdf]

nature portfolio

Corresponding author(s): Ping Mu

Last updated by author(s): Jul 29, 2022

Reporting Summary

Nature Portfolio wishes to improve the reproducibility of the work that we publish. This form provides structure for consistency and transparency in reporting. For further information on Nature Portfolio policies, see our Editorial Policies and the Editorial Policy Checklist.

Statistics

For all statistical analyses, confirm that the following items are present in the figure legend, table legend, main text, or Methods section.

n/a	Confirmed	
☐	☒	The exact sample size (*n*) for each experimental group/condition, given as a discrete number and unit of measurement
☐	☒	A statement on whether measurements were taken from distinct samples or whether the same sample was measured repeatedly
☐	☒	The statistical test(s) used AND whether they are one- or two-sided<br>*Only common tests should be described solely by name; describe more complex techniques in the Methods section.*
☐	☒	A description of all covariates tested
☐	☒	A description of any assumptions or corrections, such as tests of normality and adjustment for multiple comparisons
☐	☒	A full description of the statistical parameters including central tendency (e.g. means) or other basic estimates (e.g. regression coefficient) AND variation (e.g. standard deviation) or associated estimates of uncertainty (e.g. confidence intervals)
☐	☒	For null hypothesis testing, the test statistic (e.g. *F*, *t*, *r*) with confidence intervals, effect sizes, degrees of freedom and *P* value noted<br>*Give P values as exact values whenever suitable.*
☒	☐	For Bayesian analysis, information on the choice of priors and Markov chain Monte Carlo settings
☐	☒	For hierarchical and complex designs, identification of the appropriate level for tests and full reporting of outcomes
☐	☒	Estimates of effect sizes (e.g. Cohen's *d*, Pearson's *r*), indicating how they were calculated

Our web collection on statistics for biologists contains articles on many of the points above.

Software and code

Policy information about availability of computer code

Data collection	Benchling guide RNA designing tool was used for CRISPR guide RNA sequences. Attune Nxt software (version 4.2.1627.1) was used for collecting FACS-based competition assay data.
Data analysis	All analysis in the work was done using open-source software.<br>Bulk RNA-seq analysis was done using the QBRC Bulk RNA-seq pipeline (https://github.com/QBRC/QBRC_BulkRnaSeqDE). Alignment, quantification, and differential analysis were performed using the QBRC_BulkRnaSeqDE pipeline (https://github.com/QBRC/QBRC_BulkRnaSeqDE). Briefly, Alignment of reads to human reference genome (GRCh38, https://www.ncbi.nlm.nih.gov/assembly/GCF_000001405.26) was done using STAR (v2.7.2b)60. FeatureCounts (v1.6.4)61 was used for gene counts, biotype counts, and rRNA estimation. Differential expression analysis was performed using the R package DEseq2 (v1.26)62. Cutoff values of absolute fold change greater than 2 and FDR<0.1 were used to select for differentially expressed genes. Gene Set Enrichment Analysis (GSEA) was carried out with the R package fgsea (v1.14.0) using the 'KEGG' and 'Hallmark' libraries from MsigDB.<br>10x scRNA-seq data was preprocessed using the Cell Ranger software (5.0.0). Demultiplexing, alignment and read counting of the single-cell RNA-seq data were carried out using the 10x Genomics Cell Ranger 5.0.0. scRNA-seq data analysis was performed with the 'Scanpy' (v1.6.0) package in Python. To evaluate and model lineage plasticity as a function of cell genotype, we performed trajectory analysis using the R package 'Monocle 3'. We provided the single cell gene expression matrix containing only the highly variable genes defined as previously discussed as input and used PCA and UMAP during preprocessing steps. The trajectory was built using default parameters with the root defined from the 'loser' cluster. Custom codes for the analysis in the paper have been deposited to Github and can be accessed at https://doi.org/10.5281/zenodo.6888969.<br>Graph Pad Prism (V9.3.1, Graph Pad) is used for data graphing and statistical analysis.<br>ImageJ (version 2.0.0) was used to quantify images of organoids, migration, invasion and prostasphere formation assay.<br>FlowJo (version 10.8.0) software was used for the analysis of FACS result.

For manuscripts utilizing custom algorithms or software that are central to the research but not yet described in published literature, software must be made available to editors and reviewers. We strongly encourage code deposition in a community repository (e.g. GitHub). See the Nature Portfolio guidelines for submitting code & software for further information.

Data

Policy information about availability of data

All manuscripts must include a data availability statement. This statement should provide the following information, where applicable:

- Accession codes, unique identifiers, or web links for publicly available datasets
- A description of any restrictions on data availability
- For clinical datasets or third party data, please ensure that the statement adheres to our policy

All the described bulk and single cell RNA-seq data that support the findings of this study have been deposited in the Gene Expression Omnibus under the accession numbers GSE175975. The human CRPC tumor biopsies single cell data was downloaded from and available in the Gene Expression Omnibus (GEO) under the accession number GSE137829. Patients genomic and transcriptomic data were derived from the TCGA research network and SU2C cohort, which were queried using cbioportal (http://www.cbioportal.org/study/summary?id=prad_su2c_2019) and the Genomic Data Commons Data Portal (https://portal.gdc.cancer.gov/). SOX2 ChIP-Seq data were downloaded from and available under GSE166185. Source data for Fig. 1-6 and Extended Data Fig. 2-10 have been provided as Source Data files. Source data for Extended Fig 1 has been provided in Supplementary Table. RNA-seq Alignment of reads to human reference genome (GRCh38, https://www.ncbi.nlm.nih.gov/assembly/GCF_000001405.26/). All other data supporting the findings of this study are available from the corresponding author on reasonable request. Custom codes for the analysis in the paper have been deposited to Github and can be accessed at https://doi.org/10.5281/zenodo.6888969.

Field-specific reporting

Please select the one below that is the best fit for your research. If you are not sure, read the appropriate sections before making your selection.

☒ Life sciences ☐ Behavioural & social sciences ☐ Ecological, evolutionary & environmental sciences

For a reference copy of the document with all sections, see nature.com/documents/nr-reporting-summary-flat.pdf

Life sciences study design

All studies must disclose on these points even when the disclosure is negative.

Sample size	Sample sizes are described in the methods and/or figure legends. The selection of sample sizes were described in Methods:"Statistics & Reproducibility" section. Specifically, for all in vitro experiments, three biological triplicates were used, no statistical method was used to predetermine sample size, but our sample sizes were similar to those reported in previous studies using those cell lines. For in vivo experiments, no statistical method was used to predetermine sample size, but our sample sizes were selected based on and similar to those reported in previous studies using the same LNCaP/AR xenograft models (Arora et al., Cell 2013, PMID 24315100; Mu et al., Science 2017, PMID 28059768; Zhang et al., Cancer Cell 2020, PMID 32220301). Based on those previous studies, sample size of 8-10 tumors per group would be sufficient to detect xenograft tumor growth differences.
Data exclusions	No excluded data points
Replication	All in vitro experiments were repeated at least 2 times and achieved similar conclusions. Replicates are described in methods and/or figure legends, and the representative results are shown. All attempts at replication were successful. For in vivo experiment, the LNCaP/AR xenograft model is a highly well-credential model from which enzalutamide and apalutamide were originally discovered, demonstrating its clinical relevance (Tran et al., 2009, PMID: 19359544; Arora et al., Cell 2013, PMID 24315100; Mu et al., Science 2017, PMID 28059768; Zhang et al., Cancer Cell 2020, PMID 32220301). However, due to the time-consuming nature of this model, and budget/time limitation, in vivo xenograft studies using LNCaP/AR xenograft model were only performed once.
Randomization	All 6-7 weeks old SCID mice were purchased from Taconic Biosciences and separated into each experimental group randomly, without prior designation. For in vitro experiments, randomization of cell lines was not possible. However, all cell lines were transduced with guide RNAs either targeting the gene of interest, or non-targeting sgNT control. All comparisons were between sgGeneX and sgNT to control the covariates. All cell lines were treated and analyzed in an identical fashion and the results of three biological replicates were analyzed concurrently.
Blinding	For all in vivo studies, the tumor cell injection and followup tumor treatment was performed by one researcher, while tumor measurement and data analysis were performed by a different researcher to ensure the studies were run in a blinded manner. For all in vitro experiments, blinding is impossible as the same researcher need to treat the cells and run the analysis. However, the cell numbers were automatically measured by FACS in the competition assay or by SpectraMax iD3 automatic plate reader in cell viability assay to ensure prior knowledge of the treatment groups had no impact on results. For the quantification of migration, invasion and prostasphere assays, as well as IHC and IF staining, the pictures were coded to blind researchers to treatment or genotype groups prior to data analysis to avoid bias.

Reporting for specific materials, systems and methods

We require information from authors about some types of materials, experimental systems and methods used in many studies. Here, indicate whether each material, system or method listed is relevant to your study. If you are not sure if a list item applies to your research, read the appropriate section before selecting a response.

Materials & experimental systems

n/a	Involved in the study
☐	☒ Antibodies
☐	☒ Eukaryotic cell lines
☒	☐ Palaeontology and archaeology
☐	☒ Animals and other organisms
☒	☐ Human research participants
☒	☐ Clinical data
☒	☐ Dual use research of concern

Methods

n/a	Involved in the study
☒	☐ ChIP-seq
☐	☒ Flow cytometry
☒	☐ MRI-based neuroimaging

Antibodies

Antibodies used

For western blot, antibodies used are JAK1 (Cell Signaling Technology, Cat # 3332S, Lot#6), STAT1 (Cell Signaling Technology, Cat #9172S, Lot#26), p-STAT1(58D6) (Cell Signaling Technology, Cat #9167S, Lot#25), Rb1(4H1) (Cell Signaling Technology, Cat #5230, Lot#11), P53(DO1) (Leica Biosystems, Cat# NCL-p53-DO1,Lot#6023804), Actin(8H10D10) (Cell Signaling Technology, Cat #3700, Lot#20). JAK2(C-10) (Santa Cruz, Cat# sc-390539, Lot#C0822), JAK3 (Cell Signaling Technology, Cat# 3775, Lot#6), STAT2(D9J7L) (Cell Signaling Technology, Cat# 72604, Lot#4), STAT3(D1B2J) (Cell Signaling Technology, Cat# 30835, Lot#4), Vimentin(D21H3) (Cell Signaling Technology, Cat# 5741, Lot#8), ASCL1(EPR19840) (Abcam, Cat# ab211327, Lot#GR3368513-4), Peroxidase AffiniPure Goat Anti-Mouse IgG (H+L)(AB_10015289) (Jackson ImmunoResearch, Cat# 115-035-003), Peroxidase AffiniPure Goat Anti-Rabbit IgG (H +L)(AB_2313567) (Jackson ImmunoResearch, Cat# 111-035-003).
Dilutions of all primary antibodies are 1:1000 and secondary antibodies are 1:5000.

For ChIP-qPCR experiment, antibodies used are Sox2 (D9B8N) (Cell Signaling Technology, Cat #23064S, Lot #1), Anti-Histone H3 (acetyl K27) antibody-ChIP Grade (Abcam, Cat# ab4729, Lot# GR3357415-1) and Tri-Methyl-Histone H3 (Lys27) (C36B11) Rabbit mAb (Cell Signaling Technology, Cat #9733S, Lot #8). Dilutions of all antibodies are 1:100.

For IHC and IF antibodies used are: Jak1 (Cell Signaling Technology, Cat #3332, Lot#6), Stat1 (Cell Signaling Technology, Cat #14994, Lot#26), Stat3(D1B2J) (Cell Signaling Technology, Cat #30835, Lot#4), Alexa Fluor® 647 Anti-Cytokeratin 8(EP1628Y) (Abcam, Cat #ab192468, Lot#GR198609-1), Alexa Fluor® Anti-Cytokeratin 18(E431-1) (Abcam, Cat #ab194125, Lot#GR-200266-1), Alexa Fluor® 647 Anti-Cytokeratin 5(EP1601Y) (Abcam, Cat #ab193895, Lot#GR-219431-2), Alexa Fluor® 647 Anti-Cytokeratin 14(EP1612Y) (Abcam, Cat #ab192056, Lot#GR-201705-1), Nkx3.1(4H4) (Abcam, Cat #ab96482, Lot#GR-165836-2), PSA/KLK3(D6B1) (Cell Signaling Technology, Cat #5365, Lot#4), NDRG1(D8G9) (Cell Signaling Technology, Cat #9485, Lot#4), Vimentin(D21H3) (Cell Signaling Technology, Cat #5741, Lot#8), Synaptophysin(D8F6H) (Cell Signaling Technology, Cat #36406, Lot#1), Alexa Fluor® 647-conjugated AffiniPure Goat Anti-Mouse IgG (H+L)(AB_2338902) (Jackson ImmunoResearch, Cat# 115-605-003), Alexa Fluor® 647-conjugated AffiniPure Goat Anti-Rabbit IgG (H+L)(AB_2338078) (Jackson ImmunoResearch, Cat# 111-605-144), Donkey Anti-Mouse IgG Antibody (Biotin-SP (long spacer))(AB_2307438) (Jackson ImmunoResearch, Cat# 715-065-150), Donkey Anti-Rabbit IgG Antibody (Biotin-SP (long spacer))(AB_2340593) (Jackson ImmunoResearch, Cat# 711-065-152).
Dilutions of all primary antibodies are 1:200 except for JAK1 (1:100). Dilutions of all secondary antibodies are 1:1000

Validation

All antibodies were recommended for the specific application by the manufacturer or had previously been published for the application used in this study. Relevant validation/manufacturer/citation information:
Jak1: Cell Signaling Technology, Cat # 3332S, Lot#6; Citation: PMID: 34556638; PMID: 35024200
REACTIVITY: H M; SENSITIVITY: Endogenous; MW (kDa): 130; SOURCE: Rabbit
Application-Dilution: Western Blotting-1:1000
Jak1 Antibody detects endogenous levels of total Jak1 protein. Polyclonal antibodies are produced by immunizing animals with a synthetic peptide corresponding to residues surrounding Tyr1022/1023 of human Jak1. Antibodies are purified by protein A and peptide affinity chromatography.
https://www.cellsignal.com/products/primary-antibodies/jak1-antibody/3332
STAT1: Cell Signaling Technology, Cat #9172S, Lot#26; Citation: PMID: 34925319; PMID: 34724828
REACTIVITY: H M R Mk; SENSITIVITY: Endogenous; MW (kDa): 84, 91; SOURCE:Rabbit
Application-Dilution: Western Blotting-1:1000; Immunoprecipitation-1:50; Chromatin IP-1:50
Stat1 Antibody detects endogenous levels of total Stat1 protein. The antibody detects both Stat1alpha (91kDa) and Stat1beta (84 kDa) isoforms. Polyclonal antibodies are produced by immunizing animals with a synthetic peptide corresponding to the sequence of human Stat1. Antibodies are purified by protein A and peptide affinity chromatography.
https://www.cellsignal.com/products/primary-antibodies/stat1-antibody/9172
p-STAT1(Tyr701) (58D6): Cell Signaling Technology, Cat # 9167S, Lot#25; Citation: PMID: 34992585; PMID: 34420035
REACTIVITY: H M; SENSITIVITY: Endogenous; MW (kDa): 84, 91; Source/Isotype:Rabbit IgG
Application-Dilution: Western Blotting-1:1000; Immunoprecipitation-1:100; Immunohistochemistry (Paraffin)-1:400 - 1:1600; Immunofluorescence (Immunocytochemistry)-1:200 - 1:800; Flow Cytometry-1:100 - 1:400; Chromatin IP-1:100; Chromatin IP-seq-1:100
Phospho-Stat1 (Tyr701) (58D6) Rabbit mAb detects endogenous levels of Stat1 only when phosphorylated at tyrosine 701. The antibody detects phosphorylated tyrosine 701 of p91 Stat1 and also the p84 splice variant. It does not cross-react with the corresponding phospho-tyrosines of other Stat proteins.
https://www.cellsignal.com/products/primary-antibodies/phospho-stat1-tyr701-58d6-rabbit-mab/9167
Rb1(4H1): Cell Signaling Technology, Cat #5230, Lot#11, Citation: PMID: 8939849; PMID: 9315635; PMID: 11134518
REACTIVITY: H Mk B Pg; SENSITIVITY: Endogenous; MW (kDa): 110; Source/Isotype: Mouse IgG2a
Application-Dilution: Western Blotting-1:2000; Immunoprecipitation-1:100; Immunohistochemistry (Paraffin)-1:400 - 1:1600; Immunofluorescence (Immunocytochemistry)-1:800 - 1:3200; Flow Cytometry-1:200 - 1:800; Chromatin IP-1:200
Rb (4H1) Mouse mAb can be used in high throughput kinase assays and drug discovery applications. It detects Rb but does not recognize the Rb homologues p107 or p130. Monoclonal antibody is produced by immunizing animals with a fusion protein (Rb-C

Fusion Protein #6022) containing residues 701-928 of human Rb.
https://www.cellsignal.com/products/primary-antibodies/rb-4h1-mouse-mab/5230
P53 (DO1): Leica Biosystems, Cat# NCL-p53-DO1, Lot#6023804; Citation: PMID: 28059768;PMID: 21533191
This monoclonal antibody recognizes both wild type and mutant forms of human p53 protein under denaturing and non-denaturing conditions. The epitope recognized by clone DO-7 can be destroyed by prolonged fixation in buffered formalin. The heat induced epitope retrieval technique may improve staining in some cases.
https://shop.leicabiosystems.com/us/ihc-ish/ihc-primary-antibodies/pid-p53-protein
β-Actin (8H10D10): Cell Signaling Technology, Cat #3700, Lot#20; Citation: PMID: 35301087; PMID: 35387176
REACTIVITY: H M R Hm Mk Dg; SENSITIVITY: Endogenous; MW (kDa): 45; Source/Isotype:Mouse IgG2b
Application-Dilution: Western Blotting-1:1000; Immunohistochemistry (Paraffin)- 1:8000 - 1:32000; Immunofluorescence (Immunocytochemistry)- 1:2500 - 1:10000; Flow Cytometry (Fixed/Permeabilized) 1:200 - 1:800
β-Actin (8H10D10) Mouse mAb detects endogenous levels of total β-actin protein. Due to the high sequence identity between the cytoplasmic actin isoforms, β-actin and cytoplasmic γ-actin, this antibody may cross-react with cytoplasmic γ-actin. It does not cross-react with α-skeletal, α-cardiac, α-vascular smooth, or γ-enteric smooth muscle isoforms.
https://www.cellsignal.com/products/primary-antibodies/b-actin-8h10d10-mouse-mab/3700
Stat3(D1B2J): Cell Signaling Technology, Cat #30835, Lot#4; Citation: PMID: 35326545; PMID: 35151317; PMID: 35157848
REACTIVITY:H M R; SENSITIVITY: Endogenous; MW (kDa): 79, 86; Source/Isotype: Rabbit IgG
Application-Dilution: Western Blotting-1:1000; Immunoprecipitation-1:200; Immunohistochemistry (Paraffin)-1:100 - 1:400; Immunofluorescence (Immunocytochemistry)-1:400 - 1:800
Stat3 (D1B2J) Rabbit mAb recognizes endogenous levels of total Stat3 protein. Some unclear staining has been observed in rodent. Species reactivity for IHC-P and IF-IC is human preferred. Monoclonal antibody is produced by immunizing animals with a synthetic peptide corresponding to residues surrounding Pro695 of human Stat3 protein.
https://www.cellsignal.com/products/primary-antibodies/stat3-d1b2j-rabbit-mab/30835https://www.cellsignal.com/products/primary-antibodies/stat3-d1b2j-rabbit-mab/30835
JAK2(C-10): Santa Cruz, Cat# sc-390539, Lot#C0822; Citation: PMID: 35455080; PMID: 35265208
JAK2 (C-10) is recommended for detection of JAK2 of mouse, rat and human origin by Western Blotting (starting dilution 1:100, dilution range 1:100-1:1000), immunoprecipitation [1-2 µg per 100-500 µg of total protein (1 ml of cell lysate)], immunofluorescence (starting dilution 1:50, dilution range 1:50-1:500) and solid phase ELISA (starting dilution 1:30, dilution range 1:30-1:3000).
JAK2 (C-10) is a mouse monoclonal antibody specific for an epitope mapping between amino acids 752-780 of JAK2 of mouse origin.
https://www.scbt.com/p/jak2-antibody-c-10?requestFrom=search
JAK3: Cell Signaling Technology, Cat# 3775, Lot#6; Citation: PMID: 34286840; PMID: 32639993
REACTIVITY: H; SENSITIVITY: Endogenous; MW (kDa): 115; SOURCE: Rabbit
Application-Dilution: Western Blotting-1:1000
Jak3 Antibody detects endogenous levels of total Jak3 protein. No cross-reactivity was detected with other family members at physiological conditions. Polyclonal antibodies are produced by immunizing animals with a synthetic peptide corresponding to residues at the carboxy-terminus of Jak3. Antibodies are purified by protein A and peptide affinity chromatography.
https://www.cellsignal.com/products/primary-antibodies/jak3-antibody/3775
STAT2(D9J7L): Cell Signaling Technology, Cat# 72604, Lot#4; Citation: PMID: 34725437; PMID: 35301282
REACTIVITY:H M; SENSITIVITY: Endogenous; MW (kDa): 97, 113; Source/Isotype: Rabbit IgG
Application-Dilution: Western Blotting-1:1000; Immunoprecipitation-1:50; Immunofluorescence (Immunocytochemistry)-1:100 - 1:400; Flow Cytometry-1:50 - 1:200; Chromatin IP-1:50; Chromatin IP-seq-1:50; CUT&RUN-1:50
Stat2 (D9J7L) Rabbit mAb recognizes endogenous levels of total Stat2 protein. Monoclonal antibody is produced by immunizing animals with a synthetic peptide corresponding to residues surrounding Leu706 of human Stat2 protein.
https://www.cellsignal.com/products/primary-antibodies/stat2-d9j7l-rabbit-mab/72604
ASCL1(EPR19840): Abcam, Cat# ab211327, Lot#GR3368513-4; Citation: PMID: 31883968; PMID: 33547076
Host species: Rabbit; Suitable for: IHC-P, WB, ICC/IF, IP; Reacts with: Mouse, Human
Immunogen: Recombinant full length protein. This information is proprietary to Abcam and/or its suppliers.
https://www.abcam.com/mash1achaete-scute-homolog-1-antibody-epr19840-ab211327.html
Sox2(D9B8N): Cell Signaling Technology, Cat #23064S, Lot#1; Citation: PMID: 34934057; PMID: 34619150; PMID: 34686327
REACTIVITY: H M; SENSITIVITY: Endogenous; MW (kDa): 35; Source/Isotype: Rabbit IgG
Application-Dilution: Western Blotting-1:1000; Immunoprecipitation-1:100; Immunofluorescence (Frozen)-1:400; Immunofluorescence (Immunocytochemistry)-1:400; Flow Cytometry-1:100; Chromatin IP-1:50; Chromatin IP-seq-1:50; CUT&RUN-1:50
Sox2 (D9B8N) Rabbit mAb recognizes endogenous levels of total Sox2 protein. The abundant nonspecific cytoplasmic labeling was observed in adult brain by immunofluorescence (IF). However, the specific staining was observed in embryonic tissue, including brain, by IF. Monoclonal antibody is produced by immunizing animals with a synthetic peptide corresponding to residues surrounding Ala188 of human Sox2 protein.
https://www.cellsignal.com/products/primary-antibodies/sox2-d9b8n-rabbit-mab/23064?site-search-type=Products&N=4294956287&Ntt=23064s&fromPage=plp&_requestid=659745
Anti-Histone H3 (acetyl K27) antibody-ChIP Grade, Abcam, Cat# ab4729, Lot# GR3357415-1; Citation: PMID: 33147444; PMID: 32592040
Rabbit polyclonal to Histone H3 (acetyl K27) - ChIP Grade; Host species: Rabbit; Suitable for: ICC/IF, WB, IHC-P, ChIP, PepArr; Reacts with: Mouse, Rat, Cow, Human, Recombinant fragment
Predicted to work with: Chicken, Xenopus laevis, Arabidopsis thaliana, Drosophila melanogaster, Monkey, Zebrafish, Plasmodium falciparum, Rice, Cyanidioschyzon merolae
Immunogen
Synthetic peptide corresponding to Human Histone H3 aa 1-100 (acetyl K27) conjugated to keyhole limpet haemocyanin.
(Peptide available as ab24404)
https://www.abcam.com/Histone-H3-acetyl-K27-antibody-ChIP-Grade-ab4729.html?gclsrc=aw.ds|aw.ds&gclid=Cj0KCQjwtvqVBhCVARIsAFUxcRscatj6ayIi9g1JB61bfrTKabsO7yOQ1H0cObIwUsuUqPcd9DQwZfcaAkrLEALw_wcB
Tri-Methyl-Histone H3 (Lys27) (C36B11) Rabbit mAb, Cell Signaling Technology, Cat #9733S, Lot #8; Citation: PMID: 35399730; PMID: 35405016
REACTIVITY: H M R Mk; SENSITIVITY: Endogenous; MW (kDa): 17; Source/Isotype: Rabbit IgG
Application-Dilution: Western Blotting-1:1000; IHC-Leica® Bond™-1:200 - 1:800; Immunohistochemistry (Paraffin)-1:100 - 1:400; Immunofluorescence (Immunocytochemistry)-1:800 - 1:3200; Flow Cytometry-1:100 - 1:400; Chromatin IP-1:50; Chromatin IP-seq-1:50; CUT&RUN-1:50

Tri-Methyl-Histone H3 (Lys27) (C36B11) Rabbit mAb detects endogenous levels of histone H3 only when tri-methylated on Lys27. The antibody does not cross-react with non-methylated, mono-methylated or di-methylated Lys27. In addition, the antibody does not cross-react with mono-methylated, di-methylated or tri-methylated histone H3 at Lys4, Lys9, Lys36 or Histone H4 at Lys20. Monoclonal antibody is produced by immunizing animals with a synthetic peptide corresponding to the amino terminus of histone H3 in which Lys27 is tri-methylated.
https://www.cellsignal.com/products/primary-antibodies/tri-methyl-histone-h3-lys27-c36b11-rabbit-mab/9733?Ntk=Products&Ntt=9733&gclid=Cj0KCQjwtvqVBhCVARIsAFUxcRtK6k55Qe_aQaKRlyDOqapNftIhMm9xuW6LqlhGzwSJ7IctyCFRvVoaAqENEALw_wcB&gclsrc=aw.ds
Alexa Fluor® 647 Anti-Cytokeratin 8(EP1628Y): Abcam, Cat #ab192468, Lot#GR198609-1; Citation: PMID: 29096715
Host species: Rabbit; Conjugation: Alexa Fluor® 647. Ex: 652nm, Em: 668nm; Suitable for: ICC/IF, Flow Cyt (Intra); Reacts with: Human
Immunogen: Synthetic peptide. This information is proprietary to Abcam and/or its suppliers.
https://www.abcam.com/alexa-fluor-647-cytokeratin-8-antibody-ep1628y-ab192468.html
Alexa Fluor® Anti-Cytokeratin 18(E431-1): Abcam, Cat #ab194125, Lot#GR-200266-1; Citation: PMID: 23966837
Host species: Rabbit; Conjugation Alexa Fluor® 647. Ex: 652nm, Em: 668nm; Suitable for: Flow Cyt, ICC/IF; Reacts with: Human
Immunogen: Full length native protein (purified) corresponding to Human Cytokeratin 18.
https://www.abcam.com/cytokeratin-18-antibody-e431-1-alexa-fluor-647-ab194125.html
Alexa Fluor® 647 Anti-Cytokeratin 5(EP1601Y): Abcam, Cat #ab193895, Lot#GR-219431-2; Citation: PMID: 31839569; PMID: 33025905; PMID: 32747751
Host species: Rabbit; Conjugation: Alexa Fluor® 647. Ex: 652nm, Em: 668nm; Suitable for: Flow Cyt (Intra), ICC/IF; Reacts with: Human
Immunogen: Synthetic peptide. This information is proprietary to Abcam and/or its suppliers.
https://www.abcam.com/alexa-fluor-647-cytokeratin-5-antibody-ep1601y-ab193895.html
Alexa Fluor® 647 Anti-Cytokeratin 14(EP1612Y): Abcam, Cat #ab192056, Lot#GR-201705-1; Citation: PMID: 27762336
Host species: Rabbit; Conjugation: Alexa Fluor® 647. Ex: 652nm, Em: 668nm; Suitable for: ICC; Reacts with: Human Does not react with: Mouse, Rat
Immunogen:Synthetic peptide. This information is proprietary to Abcam and/or its suppliers.
https://www.abcam.com/alexa-fluor-647-cytokeratin-14-antibody-ep1612y-ab192056.html
Nkx3.1(4H4): Abcam, Cat #ab96482, Lot#GR-165836-2
Host species:Mouse; Suitable for: WB, ELISA, IHC-P, Flow Cyt; Reacts with: Human
Immunogen Recombinant fragment, corresponding to amino acids 1-234 of Human Nkx3.1
https://www.abcam.com/nkx31-antibody-4h4-ab96482.html
PSA/KLK3 (D6B1): Cell Signaling Technology, Cat #5365, Lot#4; Citation: PMID: 34698359; PMID: 35402240
REACTIVITY: H; SENSITIVITY: Endogenous; MW (kDa): 29; Source/Isotype: Rabbit IgG
Application-Dilution: Western Blotting-1:1000; Immunoprecipitation-1:50; Immunofluorescence (Immunocytochemistry)-1:200; Flow Cytometry-1:50
PSA/KLK3 (D6B1) XP® Rabbit mAb recognizes endogenous levels of total PSA/KLK3 protein. Monoclonal antibody is produced by immunizing animals with a synthetic peptide corresponding to residues surrounding Phe165 of human PSA/KLK3 protein.
https://www.cellsignal.com/products/primary-antibodies/psa-klk3-d6b1-xp-rabbit-mab/5365
NDRG1 (D8G9): Cell Signaling Technology, Cat #9485, Lot#4; Citation: PMID: 33499898; PMID: 33334021
REACTIVITY:H Mk; SENSITIVITY: Endogenous; MW (kDa): 46, 48; Source/Isotype: Rabbit IgG
Application-Dilution: Western Blotting-1:1000; Immunoprecipitation-1:100; Immunohistochemistry (Paraffin)-1:800; Immunofluorescence (Immunocytochemistry)-1:200
NDRG1 (D8G9) XP® Rabbit mAb recognizes endogenous levels of total NDRG1 protein. Monoclonal antibody is produced by immunizing animals with a synthetic peptide corresponding to residues near the carboxy terminus of human NDRG1 protein.
https://www.cellsignal.com/products/primary-antibodies/ndrg1-d8g9-xp-rabbit-mab/9485
Vimentin (D21H3): Cell Signaling Technology, Cat #5741, Lot#8; Citation: PMID: 35428310; PMID: 35414768
REACTIVITY: H M R Mk; SENSITIVITY: Endogenous; MW (kDa):57; Source/Isotype: Rabbit IgG
Application-Dilution: Western Blotting-1:1000; IHC-Leica® Bond™-1:200 - 1:800; Immunohistochemistry (Paraffin)-1:100 - 1:400; Immunofluorescence (Immunocytochemistry)-1:50 - 1:200; Flow Cytometry-1:50 - 1:200
Vimentin (D21H3) XP® Rabbit mAb detects endogenous levels of total vimentin protein. Monoclonal antibody is produced by immunizing animals with a synthetic peptide corresponding to residues surrounding Arg45 of human vimentin protein.
https://www.cellsignal.com/products/primary-antibodies/vimentin-d21h3-xp-rabbit-mab/5741
Synaptophysin (D8F6H): Cell Signaling Technology, Cat #36406, Lot#1; Citation: PMID: 34572962; PMID: 34671017
REACTIVITY: H M R; SENSITIVITY: Endogenous; MW (kDa): 38; Source/Isotype: Rabbit IgG
Application-Dilution: Western Blotting-1:1000; Immunohistochemistry (Paraffin)-1:200; Immunofluorescence (Frozen)-1:100
Synaptophysin (D8F6H) XP® Rabbit mAb recognizes endogenous levels of total Synaptophysin protein. Monoclonal antibody is produced by immunizing animals with a synthetic peptide corresponding to residues surrounding Gly299 of human Synaptophysin protein.
https://www.cellsignal.com/products/primary-antibodies/synaptophysin-d8f6h-xp-rabbit-mab/36406
Peroxidase AffiniPure Goat Anti-Mouse IgG (H+L)(AB_10015289): Jackson ImmunoResearch, Cat# 115-035-003 Citation: PMID: 35240918;PMID: 35017439
Based on immunoelectrophoresis and/or ELISA, the antibody reacts with whole molecule mouse IgG. It also reacts with the light chains of other mouse immunoglobulins. No antibody was detected against non-immunoglobulin serum proteins. The antibody may cross-react with immunoglobulins from other species.
Whole IgG antibodies are isolated as intact molecules from antisera by immunoaffinity chromatography. They have an Fc portion and two antigen binding Fab portions joined together by disulfide bonds and therefore they are divalent. The average molecular weight is reported to be about 160 kDa. The whole IgG form of antibodies is suitable for the majority of immunodetection procedures and is the most cost effective.
https://www.jacksonimmuno.com/catalog/products/115-035-003
Peroxidase AffiniPure Goat Anti-Rabbit IgG (H+L)(AB_2313567): Jackson ImmunoResearch, Cat# 111-035-003 Citation: PMID: 35017439;PMID: 35761940
Based on immunoelectrophoresis and/or ELISA, the antibody reacts with whole molecule rabbit IgG. It also reacts with the light chains of other rabbit immunoglobulins. No antibody was detected against non-immunoglobulin serum proteins. The antibody may cross-react with immunoglobulins from other species.
Whole IgG antibodies are isolated as intact molecules from antisera by immunoaffinity chromatography. They have an Fc portion and two antigen binding Fab portions joined together by disulfide bonds and therefore they are divalent. The average molecular weight is reported to be about 160 kDa. The whole IgG form of antibodies is suitable for the majority of immunodetection procedures and is

the most cost effective.
https://www.jacksonimmuno.com/catalog/products/111-035-003
Alexa Fluor® 647-conjugated AffiniPure Goat Anti-Mouse IgG (H+L)(AB_2338902): Jackson ImmunoResearch, Cat# 115-605-003
Citation: PMID: 35310356;PMID: 35595807
Based on immunoelectrophoresis and/or ELISA, the antibody reacts with whole molecule mouse IgG. It also reacts with the light chains of other mouse immunoglobulins. No antibody was detected against non-immunoglobulin serum proteins. The antibody may cross-react with immunoglobulins from other species.
Whole IgG antibodies are isolated as intact molecules from antisera by immunoaffinity chromatography. They have an Fc portion and two antigen binding Fab portions joined together by disulfide bonds and therefore they are divalent. The average molecular weight is reported to be about 160 kDa. The whole IgG form of antibodies is suitable for the majority of immunodetection procedures and is the most cost effective.
https://www.jacksonimmuno.com/catalog/products/115-605-003
Alexa Fluor® 647-conjugated AffiniPure Goat Anti-Rabbit IgG (H+L)(AB_2338078): Jackson ImmunoResearch, Cat# 111-605-144
Citation: PMID: 35442190; PMID: 35721135
Based on immunoelectrophoresis and/or ELISA, the antibody reacts with whole molecule rabbit IgG. It also reacts with the light chains of other rabbit immunoglobulins. No antibody was detected against non-immunoglobulin serum proteins. The antibody has been tested by ELISA and/or solid-phase adsorbed to ensure minimal cross-reaction with human, mouse and rat serum proteins, but it may cross-react with immunoglobulins from other species.
Whole IgG antibodies are isolated as intact molecules from antisera by immunoaffinity chromatography. They have an Fc portion and two antigen binding Fab portions joined together by disulfide bonds and therefore they are divalent. The average molecular weight is reported to be about 160 kDa. The whole IgG form of antibodies is suitable for the majority of immunodetection procedures and is the most cost effective.
https://www.jacksonimmuno.com/catalog/products/111-605-144
Donkey Anti-Mouse IgG Antibody (Biotin-SP (long spacer))(AB_2307438): Jackson ImmunoResearch, Cat# 715-065-150 Citation: PMID: 35402512;PMID: 35579705
Based on immunoelectrophoresis and/or ELISA, the antibody reacts with whole molecule mouse IgG. It also reacts with the light chains of other mouse immunoglobulins. No antibody was detected against non-immunoglobulin serum proteins. The antibody has been tested by ELISA and/or solid-phase adsorbed to ensure minimal cross-reaction with bovine, chicken, goat, guinea pig, syrian hamster, horse, human, rabbit and sheep serum proteins, but it may cross-react with immunoglobulins from other species.
Whole IgG antibodies are isolated as intact molecules from antisera by immunoaffinity chromatography. They have an Fc portion and two antigen binding Fab portions joined together by disulfide bonds and therefore they are divalent. The average molecular weight is reported to be about 160 kDa. The whole IgG form of antibodies is suitable for the majority of immunodetection procedures and is the most cost effective.
https://www.jacksonimmuno.com/catalog/products/715-065-150
Donkey Anti-Rabbit IgG Antibody (Biotin-SP (long spacer))(AB_2340593): Jackson ImmunoResearch, Cat# 711-065-152 Citation: PMID: 35530297;PMID: 35521515
Based on immunoelectrophoresis and/or ELISA, the antibody reacts with whole molecule rabbit IgG. It also reacts with the light chains of other rabbit immunoglobulins. No antibody was detected against non-immunoglobulin serum proteins. The antibody has been tested by ELISA and/or solid-phase adsorbed to ensure minimal cross-reaction with bovine, chicken, goat, guinea pig, syrian hamster, horse, human, mouse, rat and sheep serum proteins, but it may cross-react with immunoglobulins from other species.
Whole IgG antibodies are isolated as intact molecules from antisera by immunoaffinity chromatography. They have an Fc portion and two antigen binding Fab portions joined together by disulfide bonds and therefore they are divalent. The average molecular weight is reported to be about 160 kDa. The whole IgG form of antibodies is suitable for the majority of immunodetection procedures and is the most cost effective.
https://www.jacksonimmuno.com/catalog/products/711-065-152

Eukaryotic cell lines

Policy information about cell lines

Cell line source(s)

Parental LNCaP/AR and CWR22Pc prostate cancer cell lines were obtained from Dr. Charles Sawyers' laboratory at MSKCC5, Du145 (#HTB-81) and PC3 (#CRL-1435) cell lines were purchased from ATCC. CWR-R1 and WA01 cells were maintained in the Donald J. Vander Griend laboratory and were not directly used in this study. The existing SOX2 ChIP-Seq data generated from those two cell lines were downloaded from GSE166185. Trp53loxP/loxP, Rb1loxP/loxP murine organoids were generated from Trp53loxP/loxP, Rb1loxP/loxP mice. Human organoids were obtained from Dr. Yu Chen's laboratory at MSKCC (Tang et al., 2020,https://doi.org/10.1101/2020.10.26.355925; Mao et al., PMID: 202134417459). The organoids are cultured in 3D Matrigel according to protocol previously described (Gao et al., 2014, PMID: 25201530). Further information and requests for resources and reagents should be directed to and will be fulfilled by the corresponding author, Dr. Ping Mu (ping.mu@utsouthwestern.edu). All cell lines, plasmids and other reagents generated in this study are available from the corresponding author with a completed Materials Transfer Agreement if there is potential for commercial application.

Authentication

STR analysis (DNA fingerprinting) were performed to validate the identity of the cell lines every year and compared to ATCC cell line profiles to ensure that all the cell identities remain stable throughout the entire proposed studies. For all prostate organoids, the morphologies were assessed under microscope and their growth and mycoplasma contamination were also be tested monthly.

Mycoplasma contamination

All cell cultures were assessed for mycoplasma monthly via the highly sensitive MycoAlertTM PLUS Mycoplasma Detection kit (Lonza, Cat #LT07-710) and all results were negative.

Commonly misidentified lines
(See ICLAC register)

No commonly misidentified cell lines were used in this study.

Animals and other organisms

Policy information about studies involving animals; ARRIVE guidelines recommended for reporting animal research

Laboratory animals	Male C.B-Igh-1b/Icr Tac-Prkdcscid SCID (Severe Combined Immunodeficient) mice of 6-7 week-old were used for xenograft experiments, which were purchase from Taconic Biosciences. All animals were housed in humidity and temperature-controlled conditions with a 12h light 12h dark cycle in the pathogen free facilities at the University of Texas, Southwestern by the Animal Resource Center (ARC), and monitored closely to minimize discomfort, distress, pain or injury throughout all the course of in vivo experiments. Animal would be removed from the study and euthanized if any signs of pain and distress were detected, or the tumor volume reaches 2000mm3. The maximal tumor size was not exceeded in all reported studies.
Wild animals	This study did not use wild animals
Field-collected samples	This study did not use filed collected samples
Ethics oversight	All procedures were performed in accordance with the recommendations of the Panel on Euthanasia of the American Veterinary Medical Association and the animal protocol was approved by Institutional Animal Care and Use Committee (IACUC) of UT Southwestern Medical Center (protocol #2019-102493).

Note that full information on the approval of the study protocol must also be provided in the manuscript.

Flow Cytometry

Plots

Confirm that:

☒ The axis labels state the marker and fluorochrome used (e.g. CD4-FITC).

☒ The axis scales are clearly visible. Include numbers along axes only for bottom left plot of group (a 'group' is an analysis of identical markers).

☒ All plots are contour plots with outliers or pseudocolor plots.

☒ A numerical value for number of cells or percentage (with statistics) is provided.

Methodology

Sample preparation	For FACS-based competition assay, the LNCaP/AR cells from different genotypes were digested to single cells, then were washed with PBS by centrifugation. Then the competition cell mixture of sgTP53/RB1-RFP cells and sgNT-GFP cells were previously cultured in vitro before starting the competition assay.
Instrument	Attune NxT Acoustic Focusing Cytometer by Thermo Fisher
Software	Attune Nxt software (version 4.2.1627.1) and FlowJo software (version 10.8.0) .
Cell population abundance	The cell mixtures of sgNT-GFP and sgTP53/RB1-RFP were treated in CSS medium with 10μM enzalutamide for 8 days and the number of GFP/RFP positive cells were measured by FACS on Day0, 4 and 8. Relative cell number fold change was calculated and normalized to veh treated group as previously described (Zhang et al., 2020,PMID: 32220301).
Gating strategy	LNCaP/AR cells were first gated based on SSC-H/FSC-A FSC-H before measuring the RFP/GFP signals for the percentage of positive cells

☒ Tick this box to confirm that a figure exemplifying the gating strategy is provided in the Supplementary Information.
