# Supplementary figures and images for: Ectopic JAK–STAT activation enables the transition to a stem-like and multilineage state conferring AR-targeted therapy resistance
Source: Nat Cancer. 2022 Sep 5;3(9):1071–87. doi: 10.1038/s43018-022-00431-9 (PMC9499870; doi:10.1038/s43018-022-00431-9)

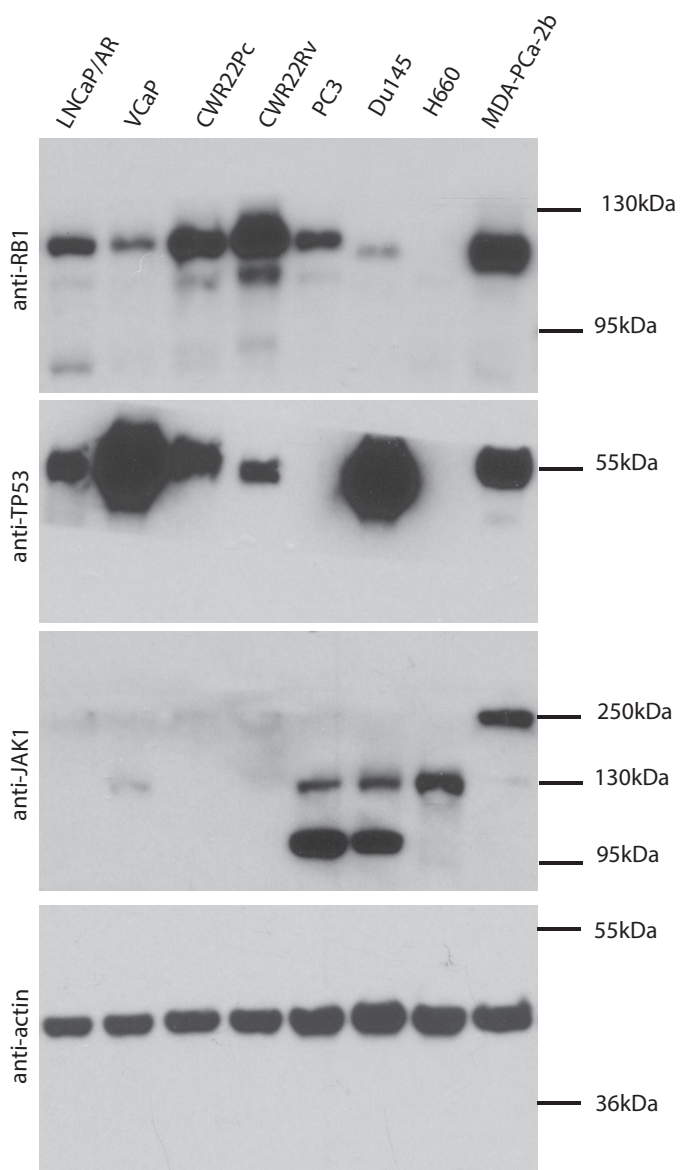

Extended Figure. 2a

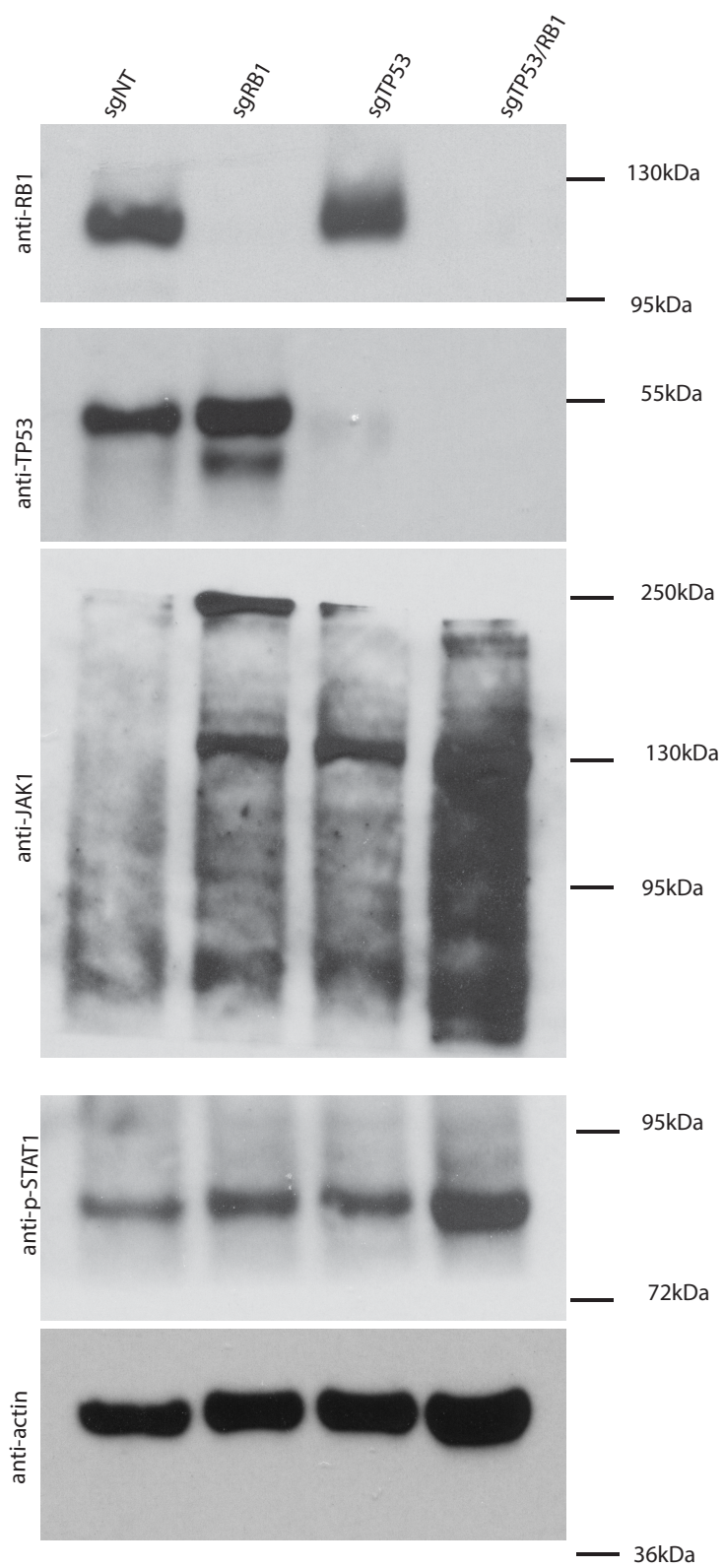

Extended Figure. 2e

Supplement: Source Data Extended Data Fig. 2 — Unprocessed blot for Extended Data Fig. 2. [file 43018_2022_431_MOESM19_ESM.pdf]

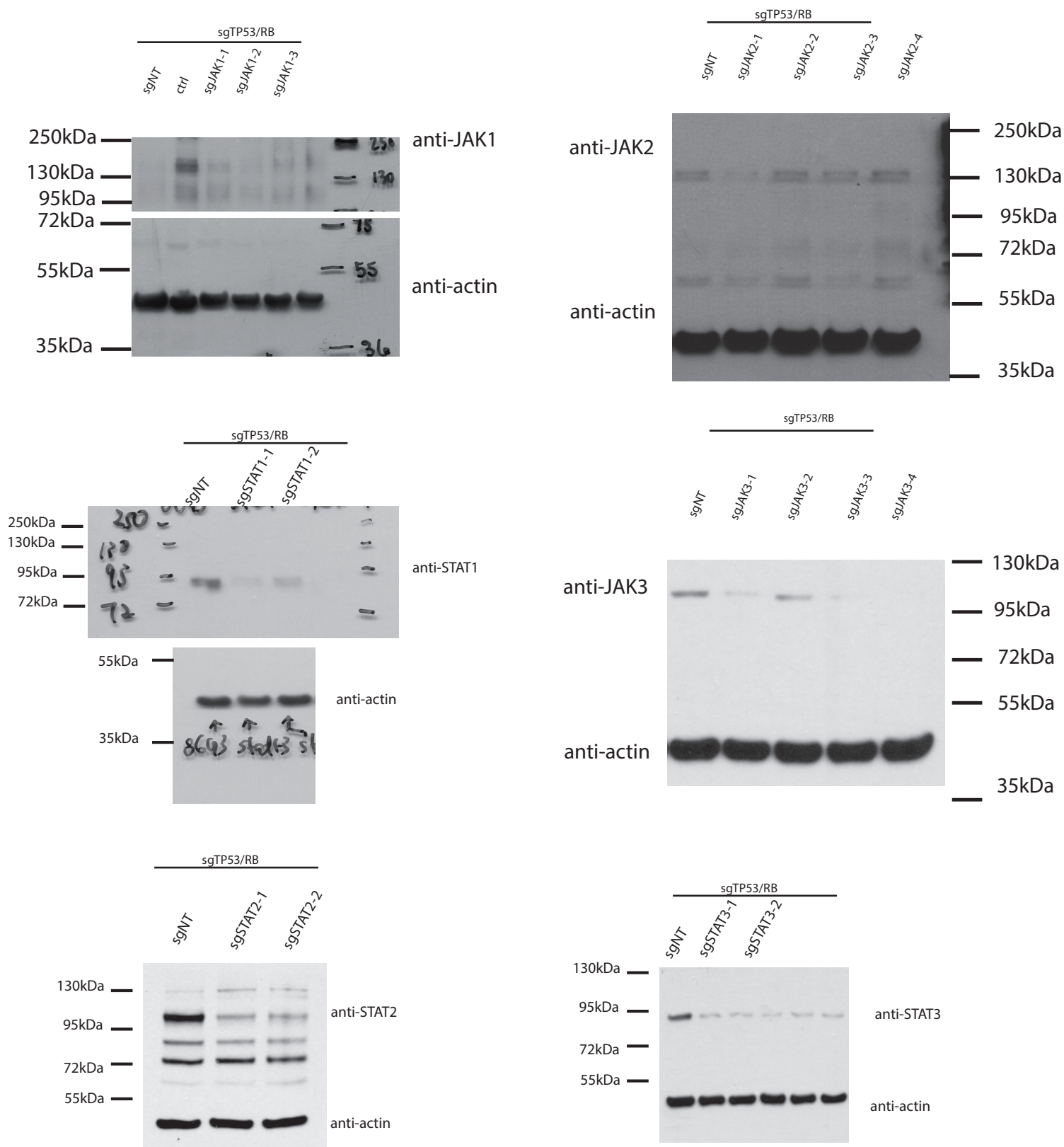

Extended Figure. 3b

Supplement: Source Data Extended Data Fig. 3. — Unprocessed blot for Extended Data Fig. 3. [file 43018_2022_431_MOESM20_ESM.pdf]

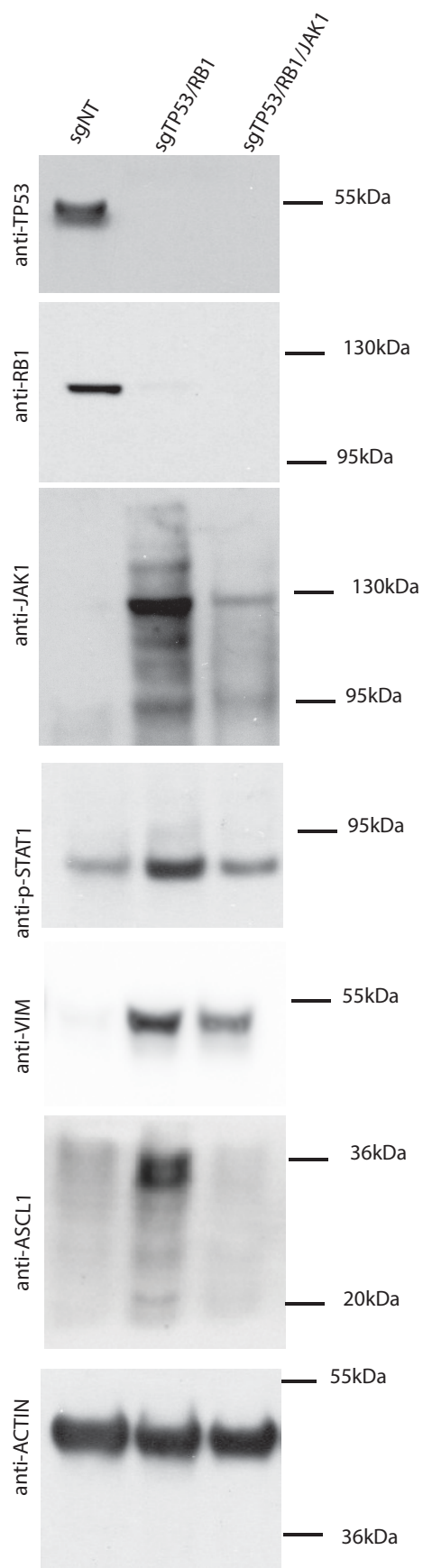

Extended Figure. 4a

Supplement: Source Data Extended Data Fig. 4 — Unprocessed blot for Extended Data Fig. 4. [file 43018_2022_431_MOESM21_ESM.pdf]
